# *Medicago Sativa* L. Saponin‐Driven *Lactobacillus Intestinalis* Restores Intestinal Stemness in Naturally Aged Mice via the Bile Acid‐FXR‐Wnt Signaling Axis

**DOI:** 10.1002/advs.202515370

**Published:** 2025-10-27

**Authors:** Mengqi Liu, Jiamin Sun, Zuyang Jia, Yalei Cui, Xiaoyan Zhu, Zhichang Wang, Hao Sun, Boshuai Liu, Yinghua Shi

**Affiliations:** ^1^ College of Animal Science and Technology Henan Agricultural University Zhengzhou Henan 450046 China; ^2^ Henan Key Laboratory of Innovation and Utilization of Grassland Resources Zhengzhou Henan 450000 China; ^3^ Henan Forage Engineering Technology Research Center Zhengzhou Henan 450002 China

**Keywords:** aging, alfalfa saponins, gut microbiota, intestinal stemness, Lactobacillus intestinalis, ursodeoxycholic acid

## Abstract

Aging is recognized as a significant risk factor for chronic diseases. The decline in intestinal stem cells function is a critical contributor to intestinal aging, resulting in impaired intestinal homeostasis and increased vulnerability to age‐related diseases. *Medicago sativa L*. (alfalfa) saponin are plant‐derived bioactive compounds that are shown to have benefits in regulating oxidative stress and gut microbiota. However, the potential of alfalfa saponin (AS) to modulate intestinal aging and enhance intestinal stemness to maintain homeostasis remains insufficiently explored. In this study, the effects of AS on intestinal stemness in naturally aged mice and its underlying mechanisms involving gut microbiota regulation are examined. Antibiotic‐mediated depletion of intestinal bacteria and fecal microbiota transplantation are employed to determine the specific role of the gut microbiota in mediating the effects of AS. Comprehensive multi‐omics analyses revealed that AS significantly increased the abundance of *Lactobacillus intestinalis* (*L. intestinalis*). Notably, *L. intestinalis* is found to possess bile acids metabolic capabilities, producing ursodeoxycholic acid, which functions as an FXR antagonist to activate the Wnt signaling pathway and enhance intestinal stemness, thereby supporting intestinal homeostasis. These findings are validated in both intestinal organoids and naturally aged mice models. This study provides the first identification of a complete functional axis by which the metabolites of AS and *L. intestinalis* modulate intestinal stemness to mitigate intestinal aging, offering insights for the development of innovative natural product‐based therapeutic strategies to promote healthy aging.

## Graphical abstract

This study examines the mechanism by which alfalfa saponin (AS) regulates intestinal stemness via modulation of the gut microbiota. The results indicate that AS significantly increases the abundance of *Lactobacillus intestinalis*, which subsequently activates Wnt signaling via bile acid metabolism and enhances intestinal stemness. These findings provide new perspectives on therapeutic strategies utilizing natural products to promote healthy aging.

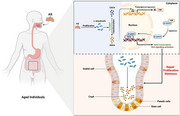

## 1 Introduction

Aging is fundamentally characterized by reduced tissue homeostasis and impaired repair mechanisms following injury. Stem cells are essential for maintaining tissue homeostasis and facilitating repair, making stem cell aging a central mechanism underlying the aging process.^[^ ^1^, ^2^ ^]^ In mammals, the intestine relies on stem cells, specifically intestinal stem cells (ISCs), which exhibit high proliferative and differentiation capacities. ISCs are primarily located in the intestinal mucosal crypts, with each crypt containing four to six ISCs at the +4 position, the fourth layer from the crypt base.^[^ ^3^ ^]^ These cells migrate from the crypt base into the intestinal lumen, where they differentiate and are eventually shed in a continuous cycle.^[^ ^4^ ^]^ In contrast to the colon, which has a turnover time of several days to weeks, mature epithelial cells in the small intestine are renewed every three to five days.^[^ ^5^ ^]^ Consequently, the small intestine is particularly susceptible to the effects of stem cell aging, with ISCs playing a critical role in maintaining intestinal homeostasis and regeneration. Aging of ISCs disrupts intestinal homeostasis, resulting in reduced digestive and absorptive functions, elevated inflammation, and degenerative tissue changes.^[^ ^6^, ^7^, ^8^ ^]^ Thus, regulation of ISCs activity is essential for preserving intestinal homeostasis.

The gut microbiota, often referred to as the “second genome” of the host, plays a critical role in maintaining physiological homeostasis.^[^ ^9^, ^10^, ^11^ ^]^ Throughout the human lifespan, the composition of the gut microbiota undergoes substantial changes, particularly during the transition to old age. Alterations in the gut microbiota of older adults are influenced by physiological changes as well as lifestyle, dietary patterns, and physical activity levels.^[^ ^12^ ^]^ In elderly individuals, the gut microbiota typically demonstrates reduced diversity, characterized by a decline in beneficial bacteria and a relative increase in potentially harmful bacteria. Specifically, beneficial species such as *Lachnospiraceae*, *Ruminococcaceae*, and *Akkermansia* are significantly reduced, while pathogenic groups like *Enterobacteriaceae* become more prevalent. The *Firmicutes* to *Bacteroidetes* (F/B) ratio also decreases, accompanied by a reduction in glycolytic bacteria and an increase in bacteria associated with protein degradation.^[^ ^13^, ^14^ ^]^ The secreted protein Amuc_1409 from *Akkermansia muciniphila* activates the Wingless‐related integration site (Wnt)/β‐catenin signaling pathway through interaction with E‐cadherin, thereby maintaining intestinal stemness in aging mice.^[^ ^15^ ^]^ Additionally, *A. muciniphila* promotes propionate metabolism to activate the Wnt signaling pathway via G protein‐coupled receptor 41 and 43, which accelerates intestinal epithelial development mediated by ISCs.^[^ ^16^ ^]^ *Alistipes senegalensis*, a recently identified anaerobic bacterial species, enhances intestinal stemness in the context of age‐related intestinal barrier damage by activating the Aryl hydrocarbon receptor signaling pathway through modulation of tryptophan metabolism, thereby facilitating intestinal barrier repair.^[^ ^17^ ^]^ Collectively, these findings indicate that intestinal homeostasis relies on a healthy microbiota, and targeted modulation of the gut microbiota may serve as an effective strategy to mitigate intestinal aging.

Natural products (NP) have always been a major driving force in drug discovery and are also an important source of anti‐aging substances today. *Medicago sativa* L. (alfalfa) belongs to the legume family and is a widely cultivated perennial multipurpose crop, extensively used for food and feed purposes.^[^ ^18^ ^]^ It contains various active compounds, including alfalfa saponins, essential oils, polysaccharides, and polyphenolic compounds.^[^ ^19^ ^]^ Saponins derived from alfalfa (AS) are one of the main active components. Our previous research found that AS can exert antioxidant effects and reduce oxidative stress in cultured cells by restoring glutathione homeostasis and acting on the mitogen‐activated protein kinase signaling pathway.^[^ ^20^, ^21^ ^]^ However, saponin molecules consist of polar sugar chains and non‐polar aglycones. The presence of the sugar chains reduces their lipophilicity, thereby limiting their solubility in water and biological membranes. Compared to other natural products such as alkaloids and flavonoids, the oral bioavailability of saponins is relatively low.^[^ ^22^ ^]^ Consequently, research on the drug metabolism of AS in animals is limited, with current reports primarily focusing on its effects on gut microbiota, which indirectly influence the host's metabolism.^[^ ^23^, ^24^ ^]^ However, it remains unknown whether AS can enhance the function of ISCs during the aging process through gut microbiota to maintain intestinal homeostasis. Therefore, further research is urgently needed to clarify the role of AS in intestinal aging.

Here, we aim to evaluate the regulatory effect of AS on intestinal stemness during the aging process and elucidate the potential molecular mechanisms involved. We found that AS can modulate the composition of the gut microbiota in naturally aging mice, increasing the abundance of *Lactobacillus intestinalis* (*L. intestinalis*) to mitigate intestinal aging. Ursodeoxycholic acid (UDCA) is a key mediator through which AS influences intestinal aging by regulating *L. intestinalis*. UDCA inhibits the Farnesoid X Receptor (FXR) signaling in intestinal epithelial cells, thereby activating the Wnt pathway. This series of changes enhances the repair capacity, proliferation, and ISCs in the intestinal crypts, promoting the homeostasis and regeneration of the intestinal epithelium. To our knowledge, this is the first identification of a functional holistic axis composed of AS, *L. intestinalis* metabolites, and intestinal stemness that reduces the impact of aging. In summary, our research provides important theoretical foundations and new insights for delaying intestinal aging and advancing healthy aging.

## 2 Results

### 2.1 AS Promotes Intestinal Epithelial Proliferation and Differentiation Mediated by ISCs in Naturally Aging Mice and Activates the Wnt Signaling pathway

**Figure 1:**
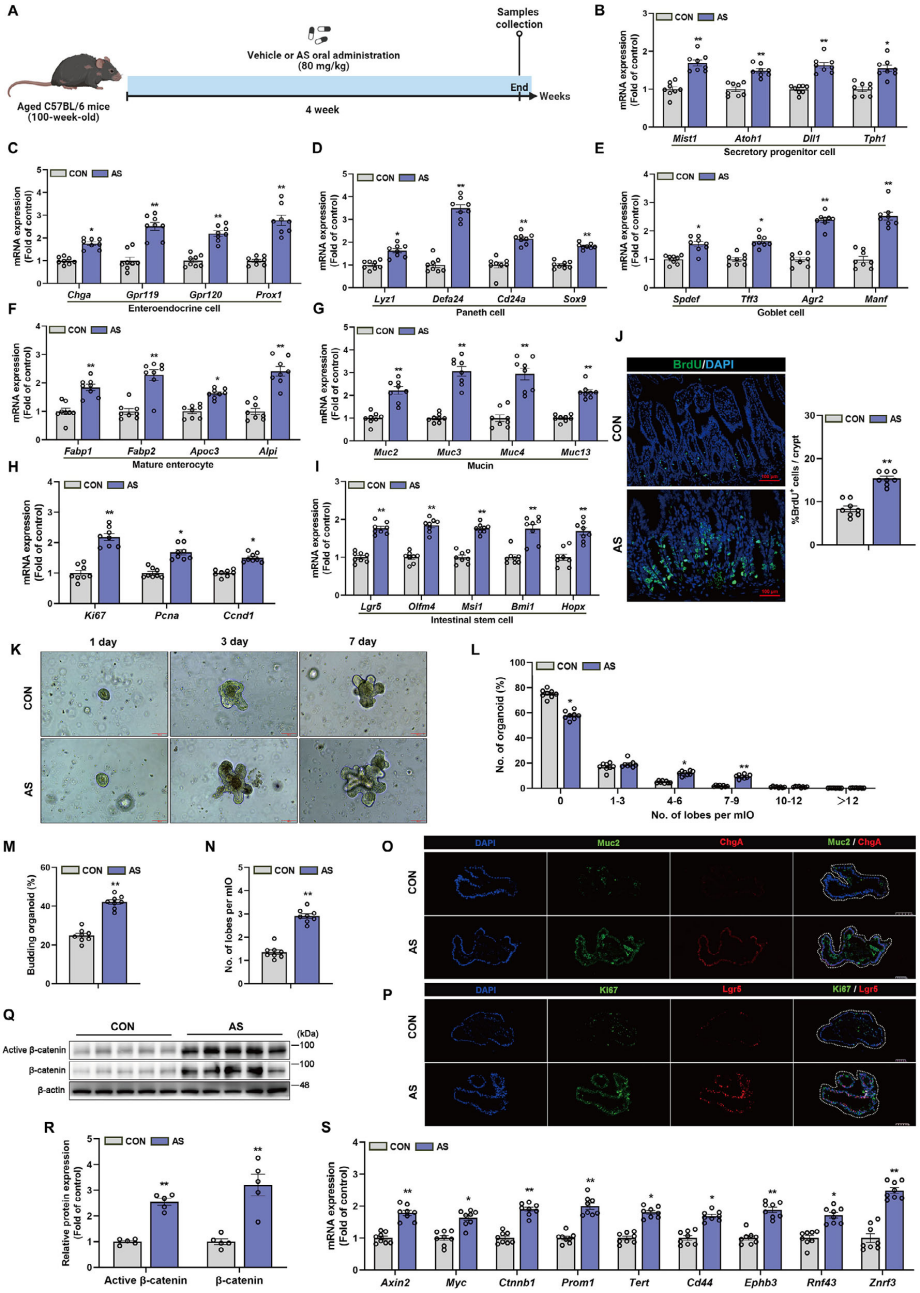
AS promotes intestinal epithelial proliferation and differentiation mediated by ISCs in naturally aged mice and activates the Wnt signaling pathway. A) Aged C57BL/6 mice (100 weeks old) were orally administered AS (80 mg kg^−1^) or vehicle control (CON) for 4 weeks. B) Relative mRNA expression levels of secretory progenitor cell markers. C) Relative mRNA expression levels of enteroendocrine cell markers. D) Relative mRNA expression levels of Paneth cell markers. E) Relative mRNA expression levels of goblet cell markers. F) Relative mRNA expression levels of mucin markers. G) Relative mRNA expression levels of mature intestinal epithelial cell markers. H) Relative expression levels of cell proliferation markers mRNA. I) Relative mRNA expression levels of ISCs markers. J) Representative immunofluorescence images of BrdU staining (left panel) and quantification of BrdU‐positive cells (green) per crypt in small intestine sections (right panel). Scale bar 100 µm. K) The organoids are derived from control group and AS group mice at 104 weeks of age after the experiment, with representative bright field images taken after 1 day, 3 days, and 7 days of culture. Scale bar 100 µm. L) Percentage distribution of organoids with specified leaf numbers. M) Average percentage of budding organoids. N) Average number of budding leaves per organoid. O) Representative images of immunofluorescence staining for the mucin marker Muc2 (green) and enteroendocrine cell marker ChgA (red) in small intestine organoids. Scale bar 100 µm. P) Representative images of immunofluorescence staining for the cell proliferation marker Ki67 (green) and stem cell marker Lgr5 (red) in small intestine organoids. Scale bar 50 µm. Q,R) Immunoblot analysis and quantification of active β‐catenin and total β‐catenin in mice small intestine tissue. S) Relative mRNA expression levels of Wnt signaling pathway target genes in mice small intestine tissue. Data were statistically analyzed using a two‐tailed Student's t‐test and presented as SEM. Statistical significance is indicated by *p < 0.05 and **p < 0.01.

The effect of AS on the differentiation and maintenance of stemness in intestinal epithelial cells in aging mice was evaluated by administering AS orally to 100‐week‐old C57BL/6 mice for four weeks (**Figure** **1**A), followed by assessment of relevant indicators. Oral administration of AS for four weeks did not result in significant intestinal or liver toxicity (Figure S1, Supporting Information). AS treatment significantly increased the transcriptional expression of genes associated with the secretory lineage and differentiation. Compared to the control group, mRNA markers for secretory progenitor cells were upregulated in the intestinal tissues of AS‐treated mice (Figure 1B). Further analysis of subtype differentiation demonstrated that mRNA expression of enteroendocrine cell markers, Paneth cell markers, and Goblet cell markers was significantly elevated in the AS group (Figure 1C–E), indicating that AS enhances the generation of various differentiated cell lineages in the intestine. AS treatment also increased mRNA expression of genes related to mature enterocytes (Figure 1F). Additionally, mucin family‐related mRNA was upregulated in the AS group (Figure 1G), supporting a positive role in differentiation associated with epithelial barrier function. Analysis of cell proliferation and ISCs activity showed that AS treatment increased mRNA expression of proliferation markers and ISCs markers (Figure 1H,I). Bromodeoxyuridine (BrdU) incorporation into newly synthesized DNA during replication, detected after injection, reflects intestinal cell proliferation activity.^[^ ^25^ ^]^ The BrdU assay confirmed that the proportion of BrdU‐positive cells in the proliferative zone of intestinal crypts was significantly higher in the AS group than in the control group, indicating enhanced ISCs proliferative activity (Figure 1J). In summary, AS enhances the multi‐lineage differentiation capacity of the intestinal epithelium in aging mice and improves ISCs activity and proliferation, thereby supporting the maintenance of intestinal structure and functional homeostasis.

To investigate the effects of AS on ISCs‐driven epithelial development, we isolated and established small intestine organoids from the intestines of two groups of naturally aged mice under different treatments. Bright‐field imaging (Figure 1K) showed that over time, the organoids in the AS group displayed more complex and branched morphologies compared to the control group. Quantitative analysis indicated a significant increase in the proportion of budding organoids in the AS treatment group, along with an increase in the average leaf count per organoid (Figure 1L–N). Furthermore, immunofluorescence staining revealed that AS treatment led to increased expression of Mucin 2 (Muc2), Chitinase‐3‐like protein 1 (ChgA), Antigen identified by monoclonal antibody Ki‐67 (Ki67), and Leucine‐rich repeat‐containing G‐protein‐coupled receptor 5 (Lgr5). This indicates that the in vitro cultured organoids also inherited the ISCs activity and enhanced epithelial complexity from the AS‐treated mice (Figure 1O,P).

The Wnt signaling pathway is essential for regulating the self‐renewal and differentiation of ISCs during intestinal homeostasis and regeneration. This pathway modulates β‐catenin activity, which is central to ISCs proliferation and differentiation. Upon binding of Wnt ligands to Frizzled receptors, the pathway is activated, resulting in the inhibition of glycogen synthase kinase 3β. This inhibition decreases β‐catenin degradation, allowing its accumulation and subsequent translocation to the nucleus. In the nucleus, β‐catenin interacts with T‐cell factor/lymphoid enhancer factor (TCF/LEF) to activate genes involved in stem cell proliferation and differentiation, including Lgr5, cellular Myelocytomatosis viral oncogene homolog, and Cyclin D1. This process supports both the proliferation of ISCs and their differentiation into various intestinal cell types.^[^ ^26^ ^]^ The present study examined the role of the Wnt pathway in AS‐mediated enhancement of ISCs proliferation and differentiation. AS treatment significantly increased the protein expression levels of β‐catenin and active β‐catenin in the intestines of aged mice, as confirmed by density analysis (Figure 1Q,R). Quantitative RT‐PCR further demonstrated significant upregulation of Wnt target gene mRNA expression (Figure 1S). These target genes are critical for cell proliferation, stem cell maintenance, tissue repair, and cellular signaling regulation, thereby supporting physiological balance and developmental processes. Collectively, these findings demonstrate that AS promotes epithelial development and multi‐lineage differentiation of ISCs in aged mice intestines by activating the Wnt/β‐catenin signaling pathway.

### 2.2 AS Reshapes the Gut Microbiota Composition of Naturally Aging Mice and Increases the Relative Abundance of *L. intestinalis*

**Figure 2:**
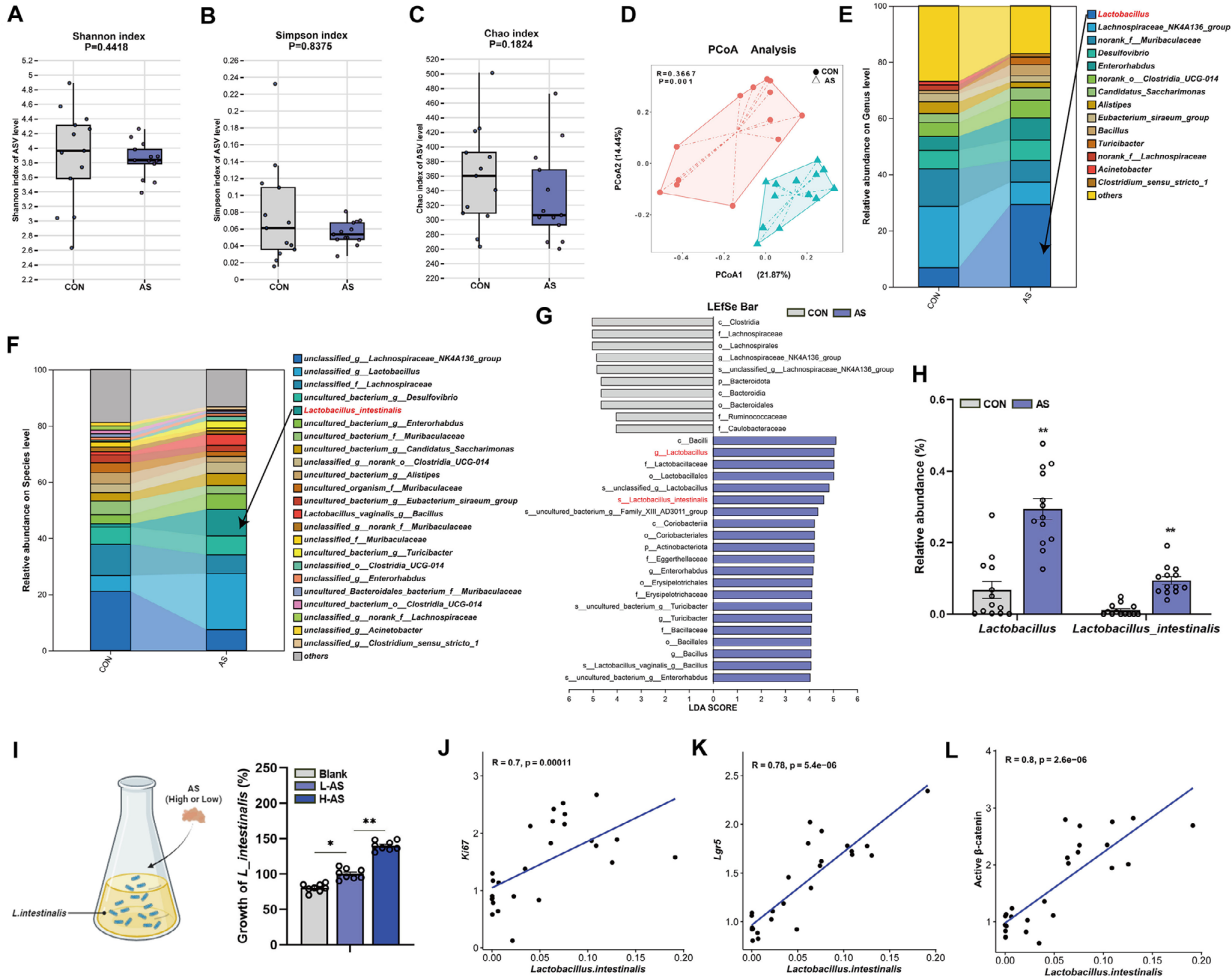
AS reshapes the gut microbiota composition of naturally aged mice and increases the relative abundance of *L. intestinalis*. A–C) Comparison of α diversity (Shannon, Simpson, and Chao indices) between the two groups. D) Principal Coordinate Analysis (PCoA) based on the Bray‐Curtis distance algorithm was used to assess the differences in microbiome composition between the CON group and the AS group, with R and p values indicated by ANOSIM analysis. E,F) Stacked bar charts of bacterial species at the genus level and species level between the two groups. G) LEfSe analysis identified significant biomarkers contributing to the differences between the two groups, with LDA > 4. H) Relative abundance of Lactobacillus and *L. intestinalis*. I) Comparison of the growth of *L. intestinalis* under different treatment conditions in the Blank, L‐AS, and H‐AS groups. J–L) Correlation analysis of *L. intestinalis* with relative mRNA expression levels of Ki67 and Lgr5 and the expression level of active β‐catenin protein. Data were statistically analyzed using a two‐tailed Student's t‐test or Mann‐Whitney U test, and are presented as mean ± SEM. Statistical significance is indicated by *p < 0.05 and **p < 0.01.

Given the critical regulatory role of gut microbiota in the aging process, 16S rRNA sequencing analysis was performed on fecal samples from mice.^[^ ^27^ ^]^ While α diversity metrics, including Shannon, Simpson, and Chao indices, showed no significant differences (**Figure** **2**A–C), β diversity principal coordinate analysis (PCoA) demonstrated clear clustering and significant separation between the control and AS groups (R = 0.3667, *p* < 0.01) (Figure 2D). This finding suggests that AS treatment significantly altered the overall composition of the gut microbiota. Further analysis identified an increased abundance of specific bacterial genera in the feces of AS group mice compared to controls. Notably, the genus Lactobacillus exhibited a significant increase in the AS treatment group (Figure 2E,F).

LEfSe analysis identified several taxa with significant differences in abundance between the two groups (LDA score > 4; Figure 2G). AS treatment significantly increased the abundance of Lactobacillus, particularly *L. intestinalis*, compared to the control group (Figure 2H). Co‐culture quantification further confirmed that both low‐dose and high‐dose AS significantly enhanced the growth of *L. intestinalis* during co‐culture compared to the blank control group (Figure 2I). These findings indicate that AS can serve as a carbon source for *L. intestinalis*, enhancing its bioactivity. This observation aligns with previous studies reporting that plant saponins act as carbon sources to promote bacterial proliferation.^[^ ^28^, ^29^, ^30^ ^]^ To determine which components of AS primarily facilitate the proliferation of *L. intestinalis*, *L. intestinalis* was co‐cultured with the 10 main components of AS. Only Soyasaponin IV, Bayogenin 3‐O‐beta‐D‐glucopyranoside, Soyasaponin II, Medicagenic acid‐3‐O‐glucopyranoside, and Soyosaponin Ac significantly promoted the proliferation of *L. intestinalis* (Figure S2, Supporting Information). Correlation analysis demonstrated a significant positive correlation between the relative abundance of *L. intestinalis* and the expression levels of the proliferation marker Ki67 (R = 0.7, p = 0.00011; Figure 2J), the ISCs marker Lgr5 (R = 0.78, p = 5.4e–06; Figure 2K), and Active β‐catenin (R = 0.8, p = 2.6e‐06; Figure 2L). *L. intestinalis* has been identified as a next‐generation probiotic.^[^ ^31^ ^]^ These findings suggest that *L. intestinalis* is a key functional bacterium in the alleviation of intestinal aging by AS. The enrichment of *L. intestinalis* may be closely related to intestinal epithelial proliferation, stem cell maintenance, and activation of the Wnt signaling pathway.

### 2.3 The Alleviation of Intestinal Aging by AS Depends on the Gut Microbiota

To determine whether the effects of AS on intestinal aging are dependent on the gut microbiota, aging C57BL/6 mice were pretreated with antibiotics (ABX) for one week to eliminate commensal gut microbiota, followed by a four‐week oral AS intervention (Figure S3A, Supporting Information). Immunofluorescence staining demonstrated that, after microbiota elimination, the number of Ki67‐positive proliferating cells and Olfactomedin 4 (OLFM4)‐positive ISCs in the AS treatment group did not significantly increase compared to the control group (Figure S3B, Supporting Information). Quantitative analysis confirmed the absence of improvement in Ki67‐positive and OLFM4‐positive cell numbers following AS intervention. Furthermore, qRT‐PCR analysis showed that AS treatment did not upregulate the expression of key ISCs marker genes (Figure S4A, Supporting Information). The expression levels of proliferation‐related genes (Figure S4B, Supporting Information) and Wnt target genes also remained unchanged after AS treatment (Figure S4C, Supporting Information).

In summary, elimination of the microbial community by ABX abolished the beneficial effects of AS on intestinal stem cell activity, epithelial proliferation, and the Wnt signaling pathway. These findings indicate that the anti‐aging effects of AS on the intestinal epithelium are dependent on the presence of symbiotic gut microbiota.

**Figure 3:**
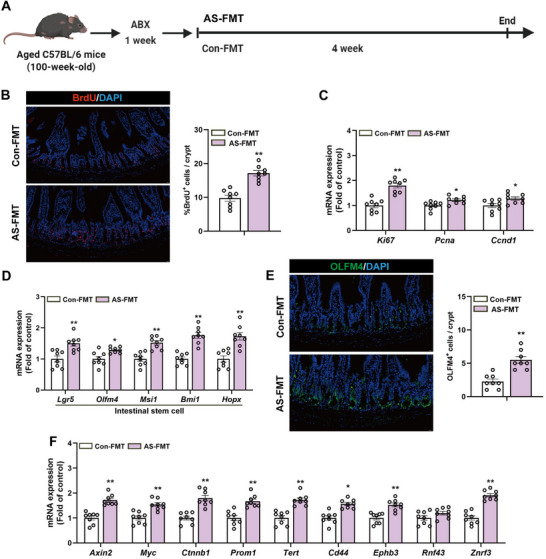
The gut microbiota mediates the effects of AS on the alleviation of intestinal aging. A) Experimental design of the treatment protocol for naturally aged C57BL/6 mice (100 weeks old), including one week of ABX treatment followed by 4 weeks of either Con‐FMT or AS‐FMT treatment. B) Representative immunofluorescence images of BrdU staining (left) and quantification of the percentage of BrdU positive cells (red) per crypt in small intestine sections (right). Scale bar 20 µm. C) Relative expression levels of the cell proliferation marker mRNA. D) Relative mRNA expression levels of ISCs markers. E) Representative images of immunofluorescence staining for the small intestine ISCs marker OLFM4 (green) along with quantitative values. Scale bar 20 µm. F) Relative mRNA expression levels of Wnt signaling pathway target genes in mice small intestine tissue. Data were statistically analyzed using a two‐tailed Student's t‐test and presented as mean ± SEM. Statistical significance is indicated by *p < 0.05 and **p < 0.01.

To determine whether the effect of AS on alleviating intestinal aging depends on the gut microbiota, the experiment utilized ABX treatment to deplete the symbiotic gut microbiota in aging mice. This was followed by fecal microbiota transplantation (FMT) from either control or AS‐treated donors (**Figure** **3**A). Immunofluorescence staining demonstrated that the AS‐FMT group had a significant increase in BrdU‐positive proliferating cells in the intestinal crypts compared to the Con‐FMT group (Figure 3B), indicating enhanced epithelial regenerative capacity after transplantation of the AS‐modified microbiota. mRNA expression analysis showed that the ISCs marker gene Ki67 and other proliferation‐related genes were significantly upregulated in the AS‐FMT group, supporting the restoration of epithelial renewal function (Figure 3C). ISCs marker gene expression was markedly higher in the AS‐FMT group than in the Con‐FMT group (Figure 3D), suggesting improved ISCs function. Immunofluorescence staining and quantitative analysis of OLFM4‐positive cells further confirmed an increased number of ISCs in the AS‐FMT group (Figure 3E). Analysis of genes associated with the Wnt signaling pathway revealed that expression of Wnt pathway target genes was significantly elevated in the AS‐FMT group compared to the Con‐FMT group (Figure 3F), indicating reactivation of the Wnt pathway.

Transplantation of gut microbiota from AS‐treated donors to ABX‐treated aged mice effectively replicates the anti‐aging effects of AS on intestinal stem cell activity, epithelial proliferation, and Wnt signaling. These findings highlight the essential role of the gut microbiota in mediating the rejuvenation of intestinal function induced by AS.

### 2.4 *L. intestinalis* Promotes the Proliferation and Differentiation of ISCs in Naturally Aging Mice by Activating the Wnt Signaling Pathway

**Figure 4:**
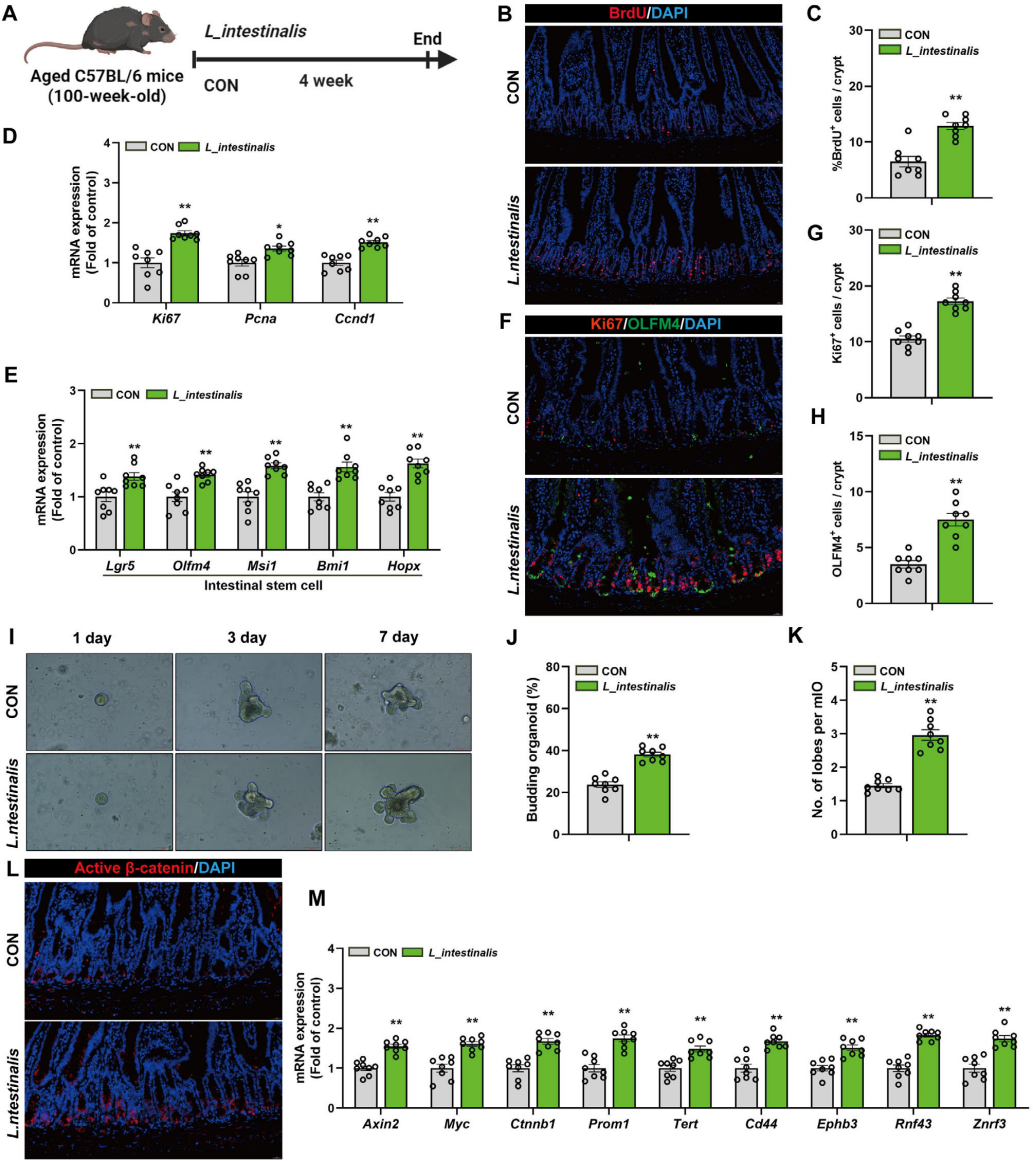
*L. intestinalis* promotes the proliferation and differentiation of ISCs in naturally aged mice by activating the Wnt signaling pathway. A) 100‐week‐old C57BL/6 mice were orally administered *L. intestinalis* (1 × 10^9^ CFU mL^−1^) or a control vehicle (CON) in a four‐week experimental study. B,C) Representative immunofluorescence images of BrdU staining and quantification of the percentage of BrdU‐positive cells (red) in each crypt of the small intestine. Scale bar, 20 µm. D) Relative expression levels of mRNA for the cell proliferation marker in intestinal tissue. E) Relative mRNA expression levels of ISCs markers in intestinal tissue. F–H) Representative images and quantification of immunofluorescence staining for the cell proliferation marker Ki67 (red) and ISCs marker OLFM4 (green) in intestinal tissue. Scale bar, 20 µm. I) Representative bright‐field images of organoids cultured from the intestines of mice in the CON group and the *L. intestinalis* group at 1, 3, and 7 days. Scale bar, 100 µm. J) Average percentage of budding organoids. K) Average number of budding leaves per organoid. L) Representative image of active β‐catenin immunofluorescence staining in mice intestinal tissue. Scale bar, 20 µm. M) Relative mRNA expression levels of target genes in the Wnt signaling pathway in mice intestinal tissue. Data were statistically analyzed using a two‐tailed Student's t‐test and presented as mean ± SEM. Statistical significance is indicated by *p < 0.05 and **p < 0.01.

To elucidate the functional role of *L. intestinalis* in regulating intestinal epithelial renewal in aging hosts, C57BL/6 mice aged 100 weeks were gavaged with *L. intestinalis* three times a week for a duration of four weeks (**Figure** **4**A). First, we employed RT‐qPCR and specific bacterial primers to determine whether *L. intestinalis* successfully colonized the small intestine of mice after oral gavage. The results showed that after oral gavage of *L. intestinalis*, there was a significant increase of *L. intestinalis* in the small intestinal contents and mucosa, indicating successful colonization in the small intestine (Figure S5, Supporting Information). Immunofluorescence analysis showed that supplementation with *L. intestinalis* significantly increased the number of BrdU‐positive proliferative cells in the intestinal crypts compared to the control group (Figure 4B,C), suggesting enhanced intestinal regenerative capacity. Correspondingly, the mRNA expression levels of proliferation markers were significantly elevated in the *L. intestinalis* group (Figure 4D). Detection of ISCs markers revealed that mRNA expression in *L. intestinalis*‐treated mice was notably higher than in the control group (Figure 4E). Immunofluorescence staining for Ki67 and OLFM4 further confirmed that the number of proliferative cells and ISCs within the crypts was significantly increased following treatment with *L. intestinalis* (Figure 4F–H).

Organoid culture experiments demonstrated that organoids derived from the *L. intestinalis* group exhibited increased branching efficiency and more complex branching morphologies during culture (Figure 4I–K), indicating enhanced activity and differentiation potential of ISCs. Immunostaining for active β‐catenin showed that the Wnt pathway activation level in the *L. intestinalis* group was significantly higher than in the control group (Figure 4L). Quantitative RT‐PCR analysis revealed upregulation of Wnt signaling target gene transcription in the intestines of mice supplemented with *L. intestinalis* (Figure 4M). Collectively, these findings indicate that oral administration of *L. intestinalis* significantly promotes ISCs proliferation and multipotent differentiation in aged mice through activation of the Wnt/β‐catenin signaling pathway.

### 2.5 Ursodeoxycholic Acid is a Key Mediator by which AS Influences Intestinal Aging through the Regulation of *L. intestinalis*

**Figure 5:**
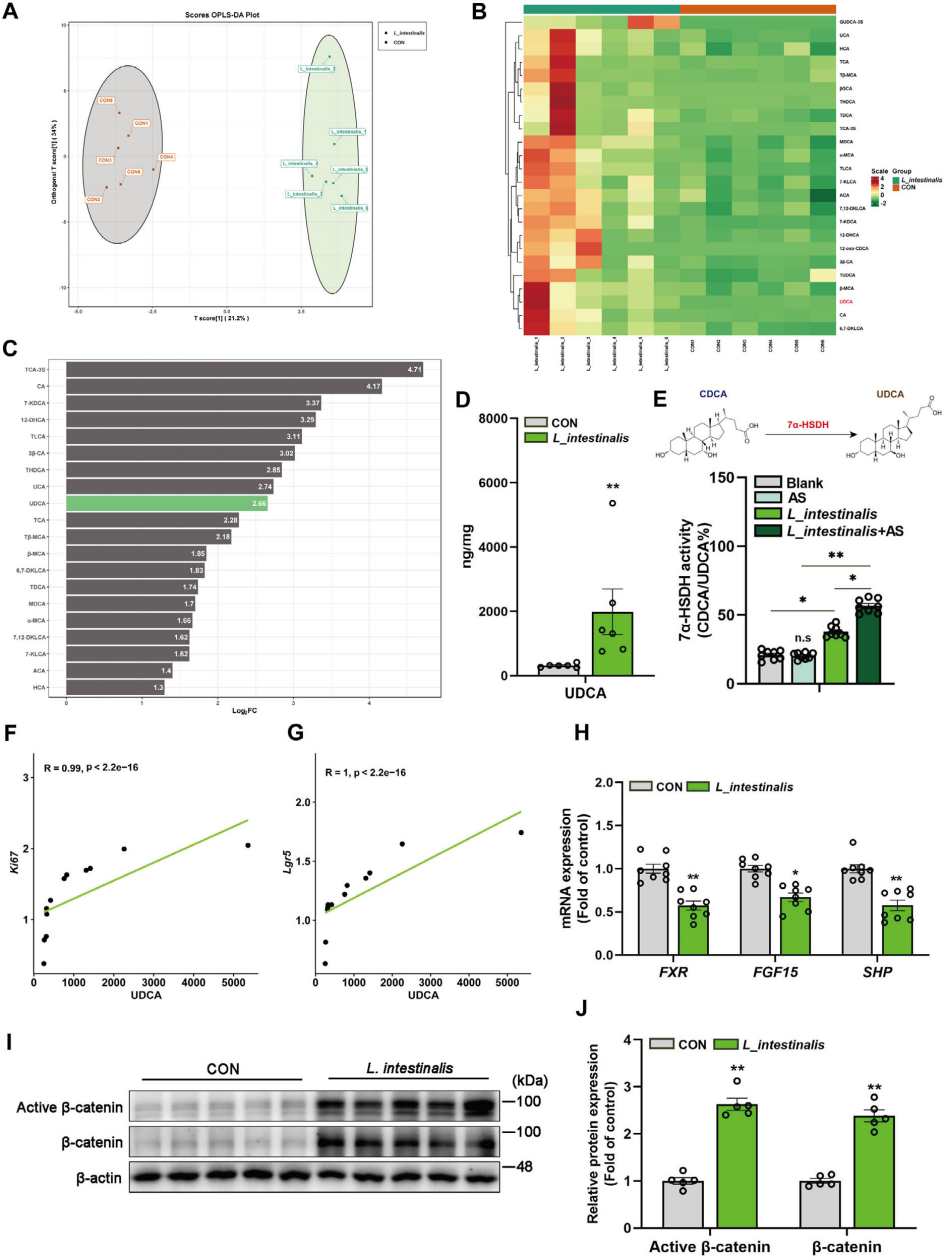
UDCA is a key mediator through which AS affects intestinal aging by regulating *L. intestinalis*. A) The OPLS‐DA score plot shows the separation between *L. intestinalis* and the control group. B) A clustered heatmap of differential BAs between *L. intestinalis* and the control group. C) Log2 fold change analysis of significantly altered metabolites, represented as a bar graph indicating effect size. D) Absolute quantitative comparison of UDCA levels in feces between the control group and the *L. intestinalis* group. E) Culturing *L. intestinalis* alone or co‐culturing *L. intestinalis* with AS promotes the production of UDCA. UDCA levels were measured to indicate 7α‐HSDH activity. F) Correlation analysis between UDCA levels and relative mRNA expression of Ki67. G) Correlation analysis between UDCA levels and mRNA expression of Lgr5. H) Relative mRNA expression levels of FXR and its target genes in intestinal tissue. I,J) Immunoblot analysis and quantification of active β‐catenin and total β‐catenin in mice small intestine tissue. Data were statistically analyzed using a two‐tailed Student's t‐test and presented as mean ± SEM. Statistical significance is indicated by *p < 0.05 and **p < 0.01.

Recent studies indicate that saponins can indirectly influence bile acids (BAs) metabolism by regulating the composition and function of the gut microbiota. This regulation promotes the growth of BAs‐metabolizing bacteria, increases deconjugation activity, and modulates the ratio of primary to secondary BAs.^[^ ^30^, ^32^ ^]^ To identify the metabolic products through which AS exerts anti‐aging effects via *L. intestinalis*, targeted BAs metabolic analysis was conducted on mice fecal samples. The OPLS‐DA score plot demonstrates a clear separation between the control and *L. intestinalis* groups (**Figure** **5**A), indicating a significant alteration in the metabolite profile. Heatmap clustering further reveals substantial differences in metabolite abundance between the groups, with several metabolites significantly upregulated in the *L. intestinalis* group (Figure 5B). The bar chart of log fold change values shows that ursodeoxycholic acid (UDCA) is among the most significantly increased metabolites in the *L. intestinalis* group (Figure 5C).

UDCA is a prominent BA whose role in modulating BAs, gut microbiota, and host metabolism has become a central focus in academic research.^[^ ^33^ ^]^ UDCA is currently the only bile acids medication approved by the U.S. Food and Drug Administration, marketed as Urso and Actigall, and is primarily indicated for the treatment of primary biliary cholangitis.^[^ ^34^ ^]^ Recent studies have demonstrated that UDCA also contributes to enhanced anti‐tumor immunity and plays a role in the prevention and treatment of SARS‐CoV‐2 infection.^[^ ^35^, ^36^ ^]^ Given its established safety profile, clinical relevance, and broad application value, UDCA was selected as the focus of this research. Quantitative analysis showed that UDCA levels in the *L. intestinalis* group were significantly higher than those in the control group (Figure 5D). When *L. intestinalis* was cultured in MRS medium supplemented with 1 g L^−1^ chenodeoxycholic acid (CDCA), the bacterium facilitated the conversion of CDCA to UDCA through the action of 7α‐hydroxy steroid dehydrogenase (7α‐HSDH), resulting in increased UDCA production. The addition of AS alone did not affect the conversion of CDCA to UDCA. However, the combined addition of AS and *L. intestinalis* further enhanced the activity of 7α‐HSDH, promoting the conversion of primary to secondary bile acids (Figure 5E). Correlation analysis identified a strong positive association between UDCA levels and the proliferation marker Ki67 (R = 0.99, *p* < 2.2e–16) as well as the intestinal stem cell marker Lgr5 (R = 1, *p* < 2.2e–16) (Figure 5F,G). RT‐qPCR analysis revealed that genes associated with the BAs receptor FXR signaling pathway, including FXR, fibroblast growth factor 15 (FGF15), and small heterodimer partner (SHP), were significantly downregulated in the *L. intestinalis* group (Figure 5H), indicating suppression of FXR signaling. Concurrently, suppression of FXR signaling was accompanied by increased protein expression of β‐catenin and active β‐catenin, suggesting activation of the Wnt signaling pathway (Figure 5I,J).

In summary, the results demonstrate that UDCA is the primary metabolic product induced by AS via the regulation of *L. intestinalis*, and it likely acts as a key mediator in alleviating intestinal aging. The findings also indicate that inhibition of FXR signaling represents a potential mechanism responsible for the enhanced intestinal regenerative capacity observed in naturally aged mice treated with AS and *L. intestinalis*.

### 2.6 UDCA Promotes the Proliferation and Differentiation of ISCs in Naturally Aging Mice and Activates the Wnt Signaling Pathway

**Figure 6:**
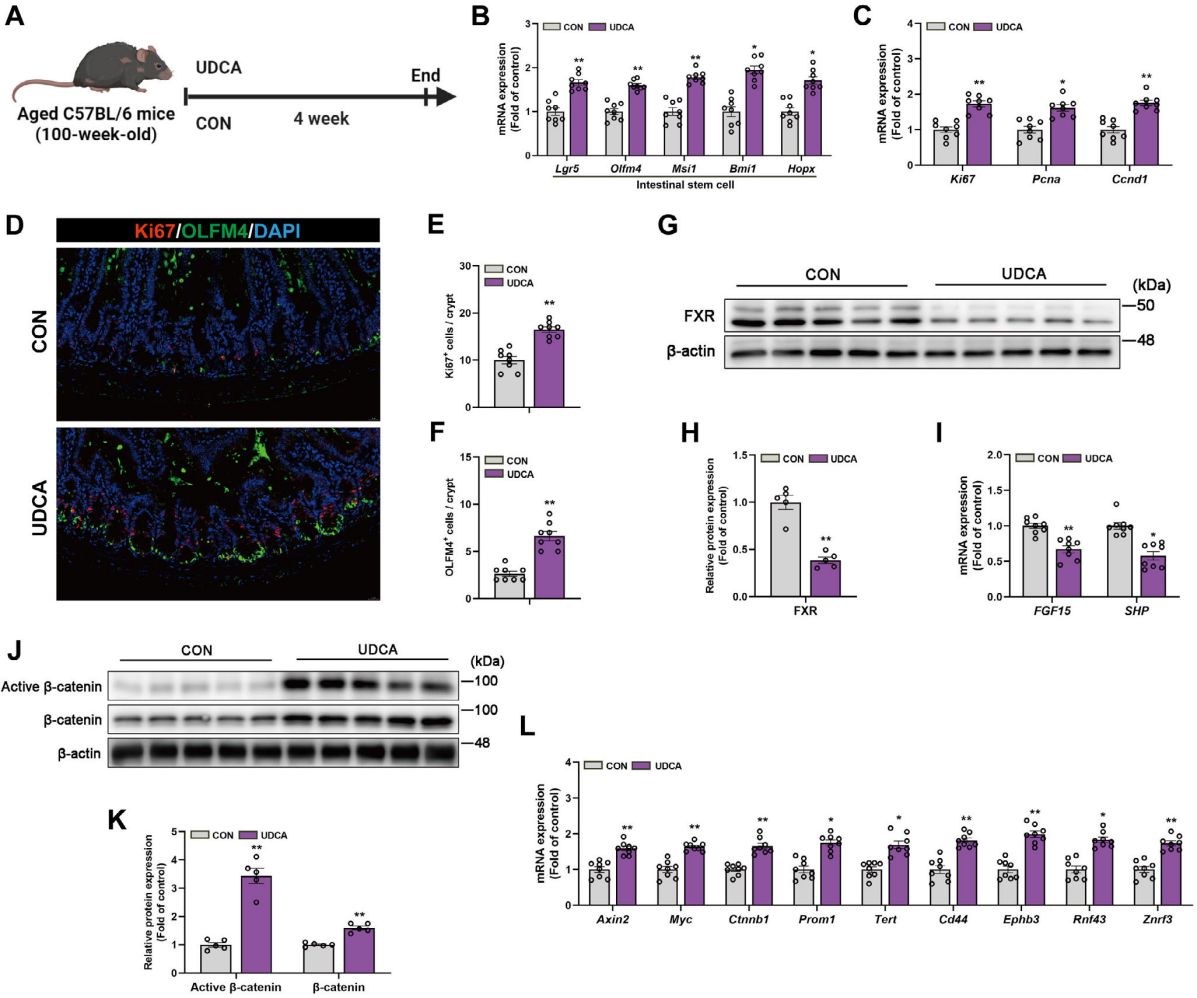
UDCA promotes the proliferation and differentiation of ISCs in naturally aged mice and activates the Wnt signaling pathway. A) Experimental design outlining the treatment protocol for aged C57BL/6 mice (100 weeks old) receiving UDCA for four weeks. B) Relative mRNA expression levels of ISCs markers. C) Relative expression levels of cell proliferation markers mRNA. D–F) Representative images and quantitative values of immunofluorescence staining for the cell proliferation marker Ki67 (red) and the ISCs marker OLFM4 (green) in the small intestine. Scale bar: 20 µm. G,H) Immunoblot analysis of FXR in mice small intestine tissue and corresponding quantitative values. I) Relative mRNA expression levels of FXR target genes in small intestine tissue. J,K) Immunoblot analysis and quantitative values for active β‐catenin and total β‐catenin in mice small intestine tissue. L) Relative mRNA expression levels of Wnt signaling pathway target genes in mice small intestine tissue. Data were statistically analyzed using a two‐tailed Student's t‐test and presented as mean ± SEM. Statistical significance is indicated by *p < 0.05 and **p < 0.01.

To validate the functional role of UDCA in mediating intestinal epithelial regeneration in aging mice, 100‐week‐old mice were administered UDCA orally every day for 4 weeks (**Figure** **6**A). qRT‐PCR analysis showed that UDCA supplementation significantly increased the mRNA levels of critical markers of ISCs (Figure 6B). Similarly, the expression of proliferation‐related markers was also significantly upregulated following UDCA treatment (Figure 6C). Immunofluorescence staining results indicated that the number of Ki67‐positive proliferative cells and OLFM4‐positive ISCs in each crypt of the UDCA group was significantly higher than that in the control group (Figure 6D–F), confirming that UDCA promotes cell proliferation and maintains stemness in vivo. Protein immunoblotting analysis showed that UDCA supplementation reduced the protein expression of the bile acids receptor FXR (Figure 6G,H), and the mRNA expression of its downstream effectors FGF15 and SHP was also significantly downregulated (Figure 6I), suggesting inhibition of the FXR signaling pathway. In terms of the Wnt signaling pathway, UDCA treatment elevated the expression levels of β‐catenin and Active β‐catenin (Figure 6J,K), and promoted the mRNA expression of Wnt target genes (Figure 6L).

In summary, the research findings demonstrate that oral UDCA enhances the activity of intestinal stem cells in aging mice, promotes their proliferation, and activates the Wnt signaling pathway. These results indicate that UDCA plays a significant role in alleviating intestinal epithelial aging and promoting tissue regeneration, potentially through mechanisms related to FXR.

### 2.7 UDCA Promotes the Proliferation and Differentiation of ISCs by Inhibiting FXR Activation of the Wnt Signaling Pathway

**Figure 7:**
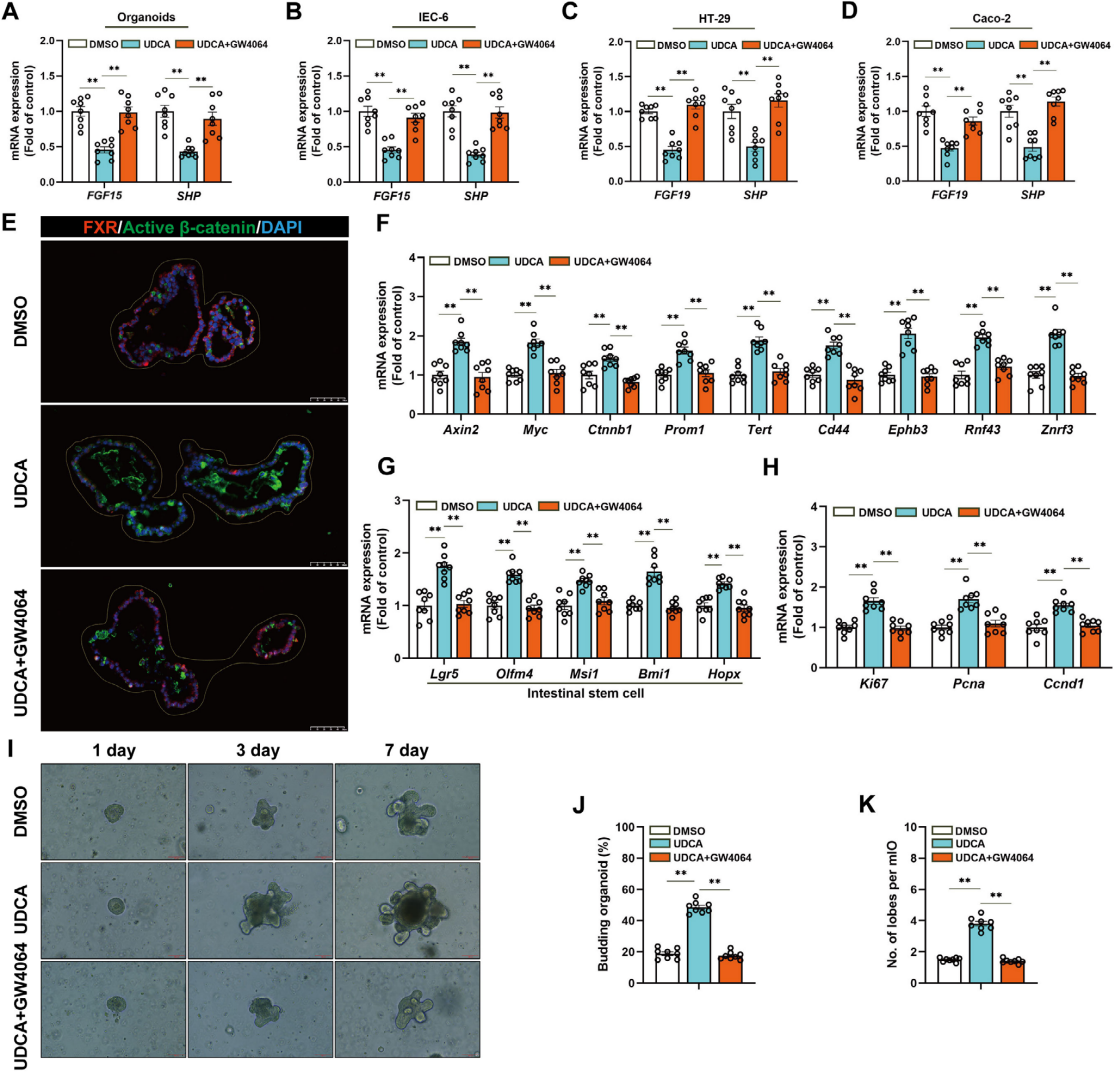
UDCA promotes the proliferation and differentiation of ISCs by inhibiting FXR activation of the Wnt signaling pathway. A–D) The relative mRNA expression levels of FGF15 (or FGF19) and SHP in different treatment groups (DMSO, UDCA, UDCA + GW4064) from organoids, IEC‐6 cells, HT‐29 cells, and Caco‐2 cells. E) Representative immunofluorescence images of FXR (red) and active β‐catenin (green) in organoids. Scale bar, 50 µm. F) The relative mRNA expression levels of Wnt signaling pathway target genes in organoids. G) The relative mRNA expression levels of ISCs markers in organoids. H) The relative expression levels of cell proliferation markers' mRNA in organoids. I) Organoids were derived from untreated blank mice aged 100 weeks after the end of the experiment, with representative bright‐field images taken after 1, 3, and 7 days of culture, 100 µm. J) The average percentage of budding organoids. K) The average number of buds per organoid. Statistical analysis of the data was conducted using one‐way analysis of variance (ANOVA) and presented as means ± SEM. Statistical significance is indicated by *p < 0.05 and **p < 0.01.

To elucidate the mechanism by which UDCA regulates ISCs function, organoids and various intestinal epithelial cell lines were treated with UDCA alone or in combination with the FXR agonist GW4064. In intestinal organoids, IEC‐6 cells, HT‐29 cells, and Caco‐2 cells, UDCA significantly downregulated the mRNA expression of FXR downstream genes fibroblast growth factor 15 (FGF15, or FGF19) and SHP. Co‐treatment with GW4064 reversed this effect (**Figure** **7**A–D). Immunofluorescence staining demonstrated that, compared to the dimethyl sulfoxide (DMSO) control group, UDCA treatment increased the protein levels of active β‐catenin in organoid cultures, indicating activation of Wnt signaling. This activation was attenuated by GW4064 (Figure 7E). UDCA also significantly upregulated the expression of Wnt signaling target genes, an effect that was markedly reduced under FXR activation by GW4064 (Figure 7F). ISCs marker genes (Figure 7G) and proliferation‐related genes (Figure 7H) exhibited a similar pattern. In organoid culture, UDCA significantly enhanced vesicle budding efficiency and the formation of branching structures, indicating increased organoid growth driven by ISCs. These effects were reversed by the addition of GW4064 (Figure 7I–K), supporting the negative regulatory role of FXR signaling in UDCA‐mediated Wnt activation and ISCs proliferation.

In conclusion, the findings demonstrate that UDCA enhances the proliferation and differentiation of intestinal stem cells primarily through inhibition of the FXR signaling pathway, which increases the epithelial regeneration capacity in aging intestines.

### 2.8 Younger Individuals have Higher Levels of *L. intestinalis* and UDCA Compared to Older Individuals

**Figure 8:**
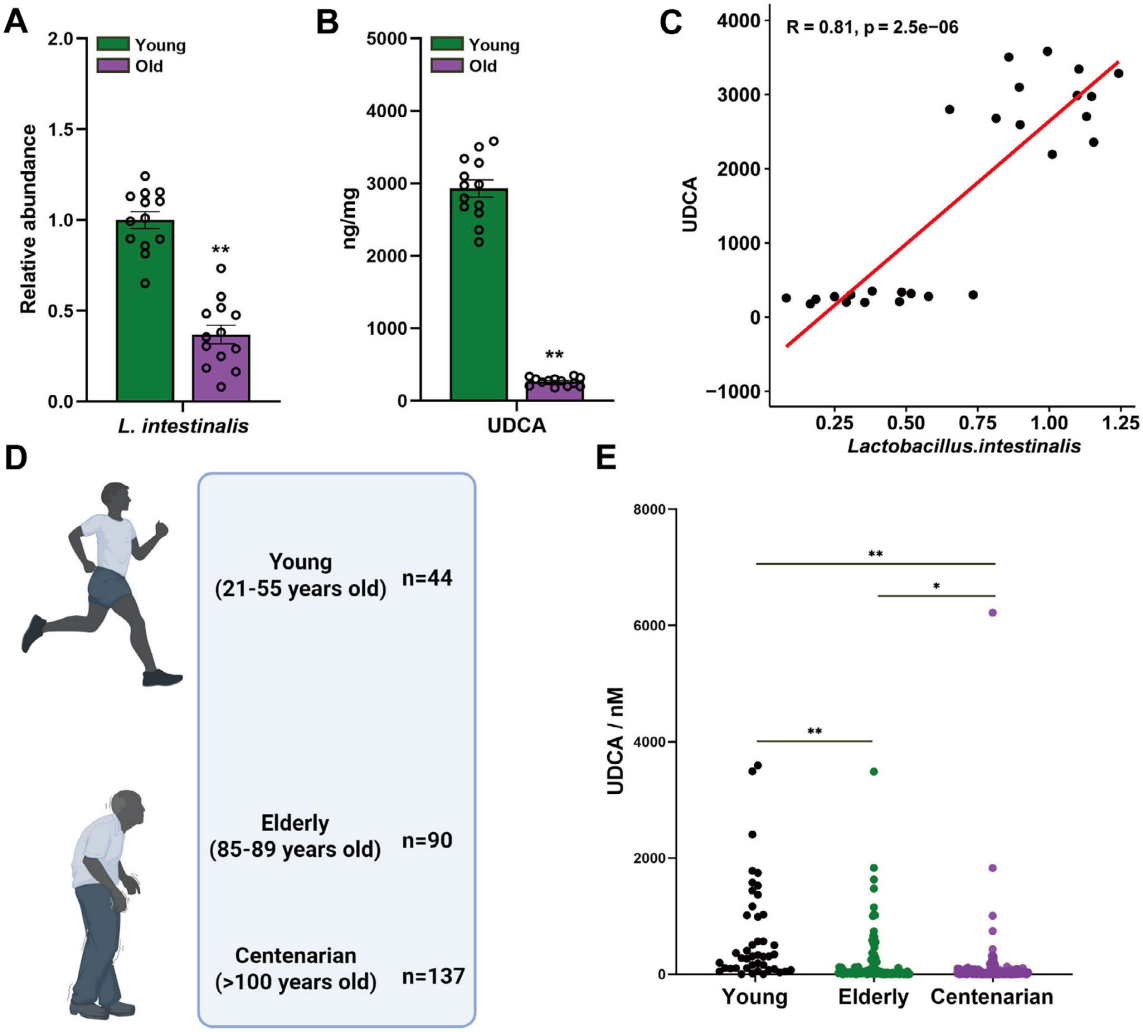
Young individuals have higher levels of *L. intestinalis* and UDCA compared to older individuals. A) The relative abundance of *L. intestinalis* in the feces of young mice (10 weeks old) and old mice. B) The levels of UDCA in the feces of young mice and old mice. C) Correlation analysis between the relative abundance of *L. intestinalis* and the levels of UDCA in the feces of young and old mice. D) Summary of participant demographics, indicating the sample size for each age group, which includes 44 young adults (ages 21–55), 90 older adults (ages 85–89), and 137 centenarians (ages >100) from Japan. E) Concentrations of UDCA in the feces of different age groups (young adults, older adults, centenarians). Data were statistically analyzed using two‐tailed Student's t‐test, Mann‐Whitney U test, or Kruskal‐Wallis test, with results expressed as mean ± SEM. Statistical significance was indicated by *p < 0.05 and **p < 0.01.

To investigate the variation of UDCA levels derived from gut microbiota with age, we first compared the relative abundance of *L. intestinalis* and fecal UDCA concentration between young and old mice. The results showed that the abundance of *L. intestinalis* in young mice was significantly higher than that in old mice (**Figure** **8**A). Correspondingly, the quantitative analysis of UDCA indicated that the UDCA content in the feces of young mice was markedly higher than that in old mice (Figure 8B). Correlation analysis revealed a significant positive correlation between *L. intestinalis* abundance and UDCA levels (R = 0.81, p = 2.5 × 10^−^⁶), further suggesting that the reduction of this strain with age is closely related to the decline in UDCA production (Figure 8C).

To evaluate the influence of human aging on this trend, publicly available metabolomics data (Metabolomics Workbench Study ID: ST001851) were analyzed to compare concentrations of UDCA in fecal samples from young adults (ages 21–55, *n* = 44), older adults (ages 85–89, *n* = 90), and centenarians (over 100 years old, *n* = 137) (Figure 8D). The concentration of fecal UDCA in young adults was significantly higher than in both the elderly and centenarian groups. Within the elderly group, fecal UDCA concentration was also significantly higher than in the centenarian group (Figure 8E). These findings indicate that, as observed in mice, UDCA concentration in the human intestine decreases with age. Previous studies have demonstrated that intestinal stemness declines with advancing human age.^[^ ^3^ ^]^ Correlation of mice and human sample data suggests that *L. intestinalis* may enhance production of the beneficial bile acids metabolite UDCA, thereby supporting intestinal stemness. Collectively, these results indicate that UDCA levels decrease in the intestines of both mice and humans with age, which may be associated with the maintenance of intestinal stemness in humans. These findings further suggest that therapies aimed at restoring UDCA levels in the elderly could potentially mitigate intestinal aging. The elevated UDCA levels observed in younger individuals may contribute to the maintenance of intestinal homeostasis and promote healthy aging.

**Figure 9:**
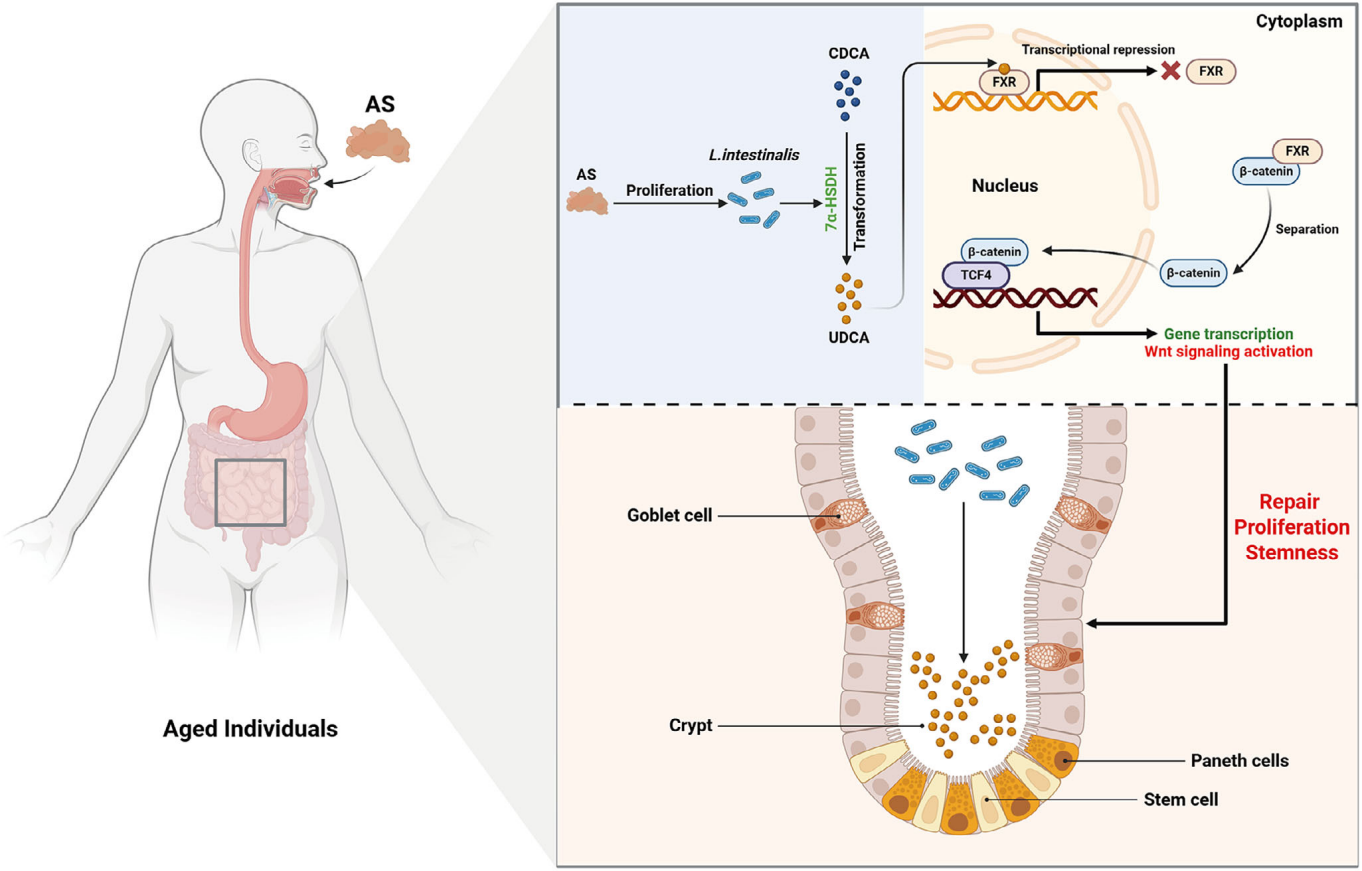
*L. intestinalis* exhibits high abundance and UDCA production capability in the intestines of naturally aged mice undergoing AS treatment. As a regulatory factor, UDCA plays a role in maintaining intestinal stemness and alleviating the progression of intestinal aging. Acting as an FXR antagonist, UDCA inhibits FXR signaling within intestinal epithelial cells, thereby activating the Wnt signaling pathway to sustain intestinal stemness. Specifically, UDCA promotes the binding of β‐catenin to TCF4 by inhibiting the transcriptional activity of FXR, which blocks the interaction between FXR and β‐catenin. This activation of the Wnt signaling pathway enhances the transcription of target genes, thereby maintaining intestinal stemness and mitigating intestinal aging.

In summary, our study explored the beneficial effects of AS on intestinal aging, finding that the gut microbiota and its metabolites play a major role (**Figure** **9**). We identified for the first time a bacterium, *L. intestinalis*, which exhibited high abundance and the ability to produce UDCA in the intestines of aged mice treated with AS. Furthermore, we established that UDCA is a regulatory factor involved in maintaining intestinal stemness and alleviating the progression of intestinal aging. UDCA acts as an FXR antagonist, inhibiting FXR signaling within intestinal epithelial cells, thereby activating Wnt signaling to maintain intestinal stemness. Overall, our research provides important theoretical foundations and new insights for utilizing natural plant extracts and probiotics to delay intestinal aging and promote healthy aging.

## 3 Discussion

As concerns about drug safety continue to intensify, research on NP‐derived drugs and functional foods is becoming increasingly important.^[^ ^37^ ^]^ Alfalfa is a widely cultivated multipurpose crop that has been extensively used as food and feed.^[^ ^18^ ^]^ Its active components exhibit various biological functions, including anti‐inflammatory and antioxidant properties. AS is one of the main bioactive components of alfalfa. In this study, we identified *L. intestinalis*, a beneficial bacterium that predominates in the intestines of aged mice treated with AS, and we discovered for the first time that *L. intestinalis* can regulate BAs metabolism, confirming its ability to enhance intestinal stemness in aged mice through the BAs‐FXR‐Wnt signaling axis.

The gut microbiota exhibits considerable complexity and diversity, shaped by genetic, environmental, and individual factors, and is closely associated with human health.^[^ ^38^, ^39^ ^]^ Multiple studies indicate that age is a major determinant of gut microbiota structure. As individuals age, the composition of the gut microbiota becomes increasingly imbalanced and its intrinsic functions decline. Recent research has identified the regulation of gut microbiota as a critical strategy for promoting health and longevity.^[^ ^12^ ^]^ In older adults, the F/B ratio in the intestines decreases significantly, which is associated with heightened susceptibility to inflammatory bowel disease.^[^ ^40^, ^41^ ^]^ Furthermore, the abundance of beneficial bacterial genera such as *Oxalobacter Allison*, *Butyricicoccus*, and *Lactobacillus* declines markedly in the elderly, while levels of pathogenic bacteria including *Escherichia coli*, *Desulfovibrio desulfuricans*, *Spinllum*, and *Proteus* increase substantially.^[^ ^42^, ^43^ ^]^ Although individual variability exists, the predominant trend is the loss of dominant core microbiota, replaced by subdominant species, which substantially elevates the risk of intestinal barrier homeostasis disruption.^[^ ^44^, ^45^ ^]^

Among symbiotic bacteria, the genus *Lactobacillus* is notable as a microaerophilic microorganism predominantly found in the gut microbiota of healthy humans. *Lactobacillus* species have demonstrated beneficial effects in conditions such as intestinal inflammation, diabetes, cancer, and neurodegenerative diseases.^[^ ^46^, ^47^, ^48^, ^49^ ^]^ Within this genus, *L. intestinalis* has recently emerged as a next‐generation probiotic. Recent studies have examined the role of *L. intestinalis* in various diseases. For example, *L. intestinalis* mediates the therapeutic effects of statins on chronic pancreatitis (CP), as statins increase the abundance of *L. intestinalis*, maintain intestinal homeostasis, inhibit bacterial migration to the pancreas, and delay CP progression by suppressing CD8+ T cell and macrophage infiltration.^[^ ^10^ ^]^ In colorectal cancer (CRC), *L. intestinalis* enhances immune cell infiltration, particularly dendritic cells, and promotes secretion of C‐C Motif Chemokine Ligand 5 from tumor cells by activating the Nucleotide‐binding oligomerization domain 1/Nuclear factor kappa‐light‐chain‐enhancer of activated B cells signaling pathway, thereby inhibiting CRC development.^[^ ^31^ ^]^ Additionally, *L. intestinalis* alleviates colitis by targeting T helper 17 cells, reducing epithelial Serum Amyloid A1 and Serum Amyloid A2 production driven by CCAAT/enhancer‐binding protein alpha, promoting retinoic acid biosynthesis, and exerting anti‐inflammatory effects.^[^ ^50^ ^]^ However, no studies have addressed the immunological and mechanistic impacts of *L. intestinalis* in the context of human aging. The present study demonstrates a decrease in the relative abundance of *L. intestinalis* in aged mice, with significant enrichment following administration of AS. Oral administration of *L. intestinalis* also enhances proliferation and stemness of ISCs in aged mice. These findings suggest that AS‐driven *L. intestinalis* plays a fundamental role in this process.

Metabolites produced by the gut microbiota function as mediator molecules that facilitate interactions between the gut microbiota and the host. These metabolites regulate immune responses, substance metabolism, tissue development, and other physiological and pathological processes in the human body.^[^ ^51^ ^]^ BAs are synthesized from cholesterol in the liver. Free bile acids are conjugated with glycine, taurine, or sulfate and subsequently released into the intestine as components of bile, where they interact with the gut microbiota. The gut microbiota can convert primary BAs into secondary BAs through bile salt hydrolase activity, dehydroxylation, and dehydrogenation, thereby driving the enterohepatic circulation of BAs and influencing systemic metabolic functions.^[^ ^52^ ^]^ Recent studies indicate that saponins can indirectly affect BAs metabolism by modulating the composition and function of the gut microbiota.^[^ ^30^, ^32^ ^]^ In the present study, *L. intestinalis* was identified as a probiotic influenced by AS, and the regulation of BAs metabolism by *L. intestinalis* has not been previously reported. This study aimed to determine whether AS regulates BAs metabolism through *L. intestinalis*. Targeted BAs metabolism analysis was performed on mice fecal samples. The results demonstrated that oral administration of *L. intestinalis* in elderly mice significantly increased levels of UDCA, a prominent secondary BAs. In vitro experiments confirmed that *L. intestinalis* promotes the conversion of CDCA to UDCA by increasing 7α‐HSDH activity, resulting in elevated UDCA levels. UDCA is recognized as a key BA, with significant attention in academic research regarding its role in the regulation of BAs, gut microbiota, and systemic metabolism.^[^ ^33^ ^]^ In this study, UDCA enhanced intestinal stemness in aging mice. Additionally, young mice exhibited a higher relative abundance of *L. intestinalis* and increased UDCA levels compared to older mice, with Spearman correlation analysis revealing a strong positive correlation between UDCA levels and *L. intestinalis* abundance. Analysis of fecal metabolites in human cohorts further showed that UDCA levels in human feces significantly decrease with age. Collectively, these findings suggest that AS regulates BAs metabolism in a *L. intestinalis*‐dependent manner, particularly through the modulation of UDCA, thereby alleviating intestinal aging. Given the observed benefits of *L. intestinalis* in enhancing intestinal stemness and promoting intestinal health in aging individuals, *L. intestinalis* may serve as a potential probiotic intervention for age‐related intestinal dysfunction. Future clinical applications could involve the use of *L. intestinalis* as a dietary supplement or therapeutic agent to restore and improve intestinal homeostasis in the elderly. Continued research into the mechanisms by which *L. intestinalis* enhances intestinal health will be essential for optimizing its clinical application and developing targeted therapies for aging‐related intestinal complications.

UDCA functions as an endogenous antagonist of FXR. FXR, a nuclear receptor, plays a central role in BAs metabolism and is predominantly distributed in organs involved in BAs processing. FXR regulates the synthesis, metabolism, and detoxification of BAs, and also contributes to immune regulation, glucose metabolism, lipid metabolism, and skeletal development.^[^ ^36^, ^53^, ^54^ ^]^ The Wnt/β‐catenin signaling pathway is highly conserved and is critical for maintaining the morphological integrity of the intestinal epithelium.^[^ ^55^ ^]^ Wnt signaling is concentrated in the crypt regions and diminishes along the crypt‐villus axis, thereby regulating ISCs activity and maintaining intestinal homeostasis.^[^ ^56^ ^]^ Previous studies have demonstrated that Wnt/β‐catenin signaling increases ISCs activity by regulating Lgr5 expression, which leads to rapid proliferation and differentiation of ISCs near sites of intestinal injury, thus facilitating reconstruction of the crypt‐villus axis.^[^ ^57^ ^]^ In the present study, FXR signaling in aged mice administered oral UDCA was significantly suppressed, whereas Wnt signaling was markedly activated. Subsequent in vitro experiments confirmed that UDCA‐mediated FXR inhibition enhanced the Wnt signaling pathway. Prior research has validated the interaction between FXR activation and Wnt signaling, specifically indicating that reduced FXR expression enhances the binding of β‐catenin to the transcription factor TCF4. When FXR does not interact with β‐catenin, nuclear accumulation of β‐catenin promotes its association with TCF4, thereby activating Wnt signaling and transcription of target genes.^[^ ^58^, ^59^ ^]^ In relation to these findings, AS increases UDCA biosynthesis by altering gut microbiota composition, notably by increasing the abundance of *L. intestinalis*. As an FXR antagonist, UDCA reduces FXR protein expression. This reduction enhances β‐catenin binding to TCF4, activating Wnt signaling and transcription of target genes, which further promotes intestinal cell proliferation and stemness. The present study provides evidence for the UDCA‐FXR‐Wnt signaling cascade as a mechanism essential for maintaining ISCs self‐renewal during aging. These results indicate that AS may serve as a novel plant‐derived bioactive compound to regulate gut microbiota, promote UDCA production, suppress FXR, and modulate the Wnt signaling pathway associated with ISCs, thereby enhancing ISCs proliferation and differentiation and mitigating intestinal aging to support healthy aging.

## 4 Limitations of the Study

This study demonstrates that AS enhances UDCA synthesis by increasing the abundance of *L. intestinalis* in the intestine, suggesting a novel mechanism to address intestinal aging. Nonetheless, several limitations should be considered. First, our findings are primarily derived from short‐term observations in mice models, which do not clarify whether AS exerts sustained effects. Future research should therefore include extended observation periods to determine the long‐term impact of AS on the gut microbiota. Although we evaluated the short‐term safety of AS in mice, the safety of prolonged use remains to be established. Addressing these limitations will improve our understanding of the interactions among the gut microbiota, BAs metabolism, and human aging. Second, while we used publicly available human metabolomic data to support the anti‐aging effects of UDCA, we could not identify relevant data on the abundance of *L. intestinalis* in human samples, which restricts the generalizability of our conclusions. Future studies should collect additional human microbiome samples to assess the distribution of *L. intestinalis* across age groups and its association with UDCA levels. In summary, future research should prioritize evaluating long‐term effects, verifying safety, and collecting more human samples to elucidate the role of AS in the aging process.

## 5 Experimental Section

### Preparation and Component Analysis of AS

AS was purchased from Baoen Biotechnology Co., Ltd. (Cangzhou, Hebei Province, China) with a purity of 85.14%. The extraction procedure was as follows: whole alfalfa plants were crushed and extracted at room temperature with 60% ethanol (10 L solvent per kg plant material, three times, 2 h each). The combined ethanol extracts were concentrated to ≈20% of the initial volume, followed by successive liquid–liquid extraction with petroleum ether, ethyl acetate, and n‐butanol. The n‐butanol fraction was loaded onto a column packed with D101 macroporous resin (Tianjin Pesticide Factory, Tianjin, China), washed with double‐distilled water until the effluent became colorless, and then eluted with 60% ethanol. The eluate was concentrated and freeze‐dried to yield a light brown powder, designated as AS. The principal constituents of AS were identified by ultra‐performance liquid chromatography coupled with quadrupole time‐of‐flight tandem mass spectrometry (UPLC‐Q‐TOF/MS). The analysis employed an Agilent Technologies 1260 ultra‐fast liquid chromatography system combined with a 6530 Accurate‐Mass Q‐Tof LC/MS system. The instrumentation included an Agilent LC‐MS system and a Thermo Fisher cooling unit. Chromatographic separation was achieved using an Ultimate UPLC XB‐C18 column (1.8 µm, 40 °C). The mobile phase consisted of acetonitrile (A) and water containing 0.1% formic acid (B), delivered at a flow rate of 0.35 mL min^−1^. A 1 µL sample, filtered through a 0.22 µm membrane, was injected to remove insoluble material. The gradient program was as follows: 0–5 min, 95% B and 5% A; 5–15 min, 15% B and 85% A; 15–30 min, 30% B and 70% A; 30–32 min, 100% B and 0% A; 32–35 min, 100% B and 0% A for column washing. Mass spectrometry was performed in positive electrospray mode with a gas temperature of 300 °C, collision energy of 20 V (automatic mode), ion mobility of 135 V, vacuum at 3.5 kV, and a drying gas flow rate of 8 L min^−1^, scanning from m/z 100 to 1800. Samples were prepared at a concentration of 512.36 mg, diluted to 5 mL with 50% acetonitrile, and mixed thoroughly prior to analysis. Results are provided in Figure S6 (Supporting Information) and Table S1 (Supporting Information).

### Ethical Statement

Animal experiments received approval from the Animal Care and Use Committee of Henan Agricultural University, China (Approval No. HENAU‐2024‐0117).

### Human Sample Data

To further validate the results, concentrations of ursodeoxycholic acid were retrieved and reanalyzed from the publicly available Metabolomics Workbench database across different age group cohorts (Study ID: ST001851). The datasets comprised 44 young adults (ages 21 to 55), 90 elderly individuals (ages 85 to 89), and 137 centenarians (over 100 years old) from Japan.^[^ ^60^ ^]^

### Animal Experiments

C57BL/6 mice (Purchased from Henan Skebes Biotechnology Co., Ltd.) aged 100 weeks were housed in a specific pathogen‐free facility, with the environmental temperature controlled between 20–22 °C and humidity maintained at 40–60%. A 12‐h light/12‐h dark cycle was implemented, and standard laboratory rodent chow and filtered water were provided. All mice were randomly assigned to experimental groups, with no differences in body weight observed prior to treatment.

### Oral AS Experiment

Mice (*n* = 14) received either phosphate‐buffered saline (PBS) as a carrier or AS at a dose of 80 mg kg^−1^ body weight by oral gavage once daily for four weeks. Fecal samples were collected each day during the final week of the experiment for subsequent fecal microbiota transplantation (FMT) trials.

### Antibiotic Experiment

Mice (*n* = 14) received ABX treatment in their drinking water for one week to eradicate gut microbiota. The ABX mixture consisted of vancomycin (0.5 mg mL^−1^, Cat: V8050, Solarbio, China), metronidazole (1 mg mL^−1^, Cat: M8060, Solarbio, China), kanamycin (1 mg mL^−1^, Cat: K8020, Solarbio, China), and ampicillin (1 mg mL^−1^, Cat: A8180, Solarbio, China). ABX‐treated mice were housed in sterile cages and provided with sterile food and water. All handling procedures were conducted under sterile conditions. After ABX treatment, mice received either vehicle (PBS) or AS for four weeks.

### FMT Experiment

Mix 600 mg of fecal sample with 6 mL of PBS quickly and evenly, then centrifuge at 1000 × g to collect the supernatant. To prevent the influence of oxygen on anaerobic bacteria, the entire procedure should be carried out in an anaerobic chamber or a nitrogen‐filled container. After one week of ABX treatment, mice were treated with Con‐FMT or AS‐FMT for four weeks. As previously described, 200 µL of the bacterial suspension was transferred to each recipient mice via intragastric administration within a time frame of 10 min.^[^ ^9^ ^]^

### Oral Bacteria Experiment

*L. intestinalis* (B293738, the same as ATCC 49 335, purchased from Ningbo Mingzhou Biotechnology in China) was cultured in MRS medium under microaerobic conditions at 37 °C. Mice (*n* = 14) were administered the vehicle (PBS) or *L. intestinalis* (1 × 10^9^ colony‐forming units (CFU) mL^−1^) for 4 weeks (200 µL each time, three times a week) via oral gavage.^[^ ^10^ ^]^

### Oral UDCA Experiment

Mice (*n* = 14) were subjected to daily oral gavage with either a vehicle (PBS) or UDCA (50 mg kg^−1^ BW, MCE #HY‐13771, USA) once a day for four weeks.^[^ ^11^ ^]^ During sampling, the experimental mice were euthanized using a carbon dioxide chamber, and samples were taken from the small intestine, liver, and serum. A portion of the small intestine sample was fixed in 4% formaldehyde for histological analysis, while the remaining samples of the small intestine, liver, serum, and feces were stored at −80 °C for future analysis.

### Enzyme‐Linked Immunosorbent Assay

Diamine oxidase (DAO), D‐lactic acid (D‐LA), lipopolysaccharide (LPS), interleukin‐6 (IL‐6), tumor necrosis factor‐alpha (TNF‐α), alanine aminotransferase (ALT), aspartate aminotransferase (AST), malondialdehyde (MDA), superoxide dismutase (SOD), and glutathione (GSH) levels in mice serum and small intestine tissue were quantified using ELISA kits according to the manufacturer's instructions (Shanghai Enzyme‐linked Biotechnology Co. Ltd., Shanghai, China).

### Real‐Time Quantitative PCR

Tissue samples collected from mice were rapidly frozen in liquid nitrogen and stored at −80 °C until analysis. Total RNA was isolated using AG RNAex Pro RNA (Code No. AG21101, Accurate Biotechnology Co., Ltd, China) following the manufacturer's protocol. RNA quality and concentration were measured with a NanoDrop spectrophotometer. For complementary DNA (cDNA) synthesis, 2 µg of total RNA was reverse transcribed using EVO M‐MLV RT Premix for qPCR (Code No. AG11706, Accurate Biotechnology Co., Ltd, China) according to the manufacturer's instructions. mRNA expression was quantified using the SYBR Green Premix Pro Tag HS qPCR Kit (Code No. AG11701, Accurate Biotechnology Co., Ltd, China) on the Roche LightCycler 96 system (Basel, Switzerland) under the recommended amplification conditions. Relative mRNA expression was normalized to the reference gene β‐actin. Primer sequences for all analyzed genes are listed in Table S2 (Supporting Information). Relative mRNA expression was calculated using the 2−ΔΔCt method. All reactions were performed in triplicate, and data analysis was conducted using the Roche LightCycler 96 system software.

### BrdU Treatment

To perform BrdU staining, 200 µL of BrdU solution (5 mg mL^−1^; Cat: B8010, Solarbio, China) in PBS was administered via intraperitoneal injection 6 h before mice euthanasia.

### Organoid Isolation and Culture

Longitudinally dissect the small intestine of the mice and place it in a dish containing cold PBS. Wash using a vortex mixer, then cut the tissue into small pieces of 1–2 mm. Using a 10 mL serological pipette pre‐wetted with PBS, gently pipette up and down ten times. Allow the tissue to settle under gravity for ≈30 s, discard the supernatant, and add 10 mL of cold PBS. Repeat this process 20–25 times. Transfer the tissue fragments into a gentle cell dissociation reagent (bioGenous, Suzhou, China) and incubate at room temperature on a shaker for 20 min. After gravity sedimentation, discard the supernatant, and add 10 mL of cold PBS containing 0.1% (w/v) bovine serum albumin (BSA). Use a 10 mL serological pipette to aspirate up and down, then filter the supernatant through a 70 µm cell strainer to collect the intestinal crypt components. Assess the purity of the crypts under a microscope, select the portions rich in intestinal crypts, and centrifuge at 270 × g for 5 min at 4 °C. Discard the supernatant, resuspend the pellet in 10 mL of high‐performance DMEM/F‐12 medium (Thermo Fisher Scientific) for washing, and centrifuge again at 270 × g for 5 min at 4 °C. Mix the pelleted crypts with Matrigel (M315066, bioGenous, Suzhou, China) and seed 50 µL into pre‐warmed 24‐well culture plates. Allow the Matrigel to polymerize for 20 min, then add 500 µL of crypt culture medium (K2001‐MI, bioGenous, Suzhou, China) to each well. Place the culture plates in a humidified incubator at 37 °C with 5% CO2. After 7 days of culture, subculture the organ samples. For each 24‐well culture plate, seed at least 50 crypt fragments for the experiment. Organoids will be exposed to UDCA (20 µm, MCE #HY‐13771, USA) with or without GW4064 (1 µm, MCE #HY‐50108, USA) for a treatment duration of 7 days. Following the exposure, RNA would be extracted using TRIzol.

### Immunofluorescence Staining

For small intestine or organoid samples, tissue sections of 5 µm were dewaxed and rehydrated after fixation, followed by heat retrieval or enzymatic treatment to enhance antigen accessibility. Next, the sections were treated with a blocking solution to reduce non‐specific binding, and a primary antibody diluted to an appropriate concentration was added, incubating overnight at 4 °C. After washing, a secondary antibody labeled with a fluorescent dye and HRP was added, incubated at room temperature for 1–2 h, followed by another wash to remove unbound secondary antibody. Finally, the cell nuclei were stained with 4′,6‐diamidino‐2‐phenylindole (DAPI) (C0065; Solarbio, China), and fluorescence images were captured using a Nikon Eclipse TI‐SR fluorescence microscope and Nikon DS‐U3 imaging system. The antibodies used in this study are detailed in Table S3 (Supporting Information).

### Western Blot

Add 900 µL of RIPA lysis buffer (P0013B; Beyotime, Shanghai, China) containing 1% protease inhibitor (P6730; Solarbio, China) and 1% phosphatase inhibitor mixture (P1260; Solarbio, China) to the sample, and grind thoroughly to disrupt the tissue at 4 °C. Next, centrifuge at 14000 × g for 15 min at 4 °C to collect the supernatant for Western blot analysis. After extracting total protein, determine the protein concentration using a BCA protein assay kit (PC0020; Solarbio, China), standardize the concentration, boil the protein, and separate it using 10% polyacrylamide gel for sodium dodecyl sulfate‐polyacrylamide gel electrophoresis (SDS‐PAGE). Then, transfer the proteins to a 0.45 µm polyvinylidene fluoride (PVDF) membrane (Millipore; Billerica). Following this, block the membrane at room temperature with 1x Tris‐buffered saline‐Tween (TBST) containing 5% nonfat dry milk for 1 h, and then incubate overnight at 4 °C with the primary antibody. After overnight incubation with the specific primary antibody, wash the membrane three times with 1x TBST for 10 min each time, and then incubate at room temperature with horseradish peroxidase (HRP)‐labeled IgG for 2 h. After incubation, wash the membrane again three times with 1x TBST for 10 min each time. Finally, prepare the chemiluminescent substrate (P0018FM; Beyotime, Shanghai, China) in a 1:1 ratio to capture the bands. Use Image J v1.8.0 software to analyze the grayscale values of the target bands, calculating the expression level of the target protein by dividing the grayscale value of the target protein by that of the internal control. The relative protein level of the control group was defined as 100%. Each sample was tested in triplicate.

### 16S rRNA Gene Sequencing

Sequencing and analysis of 16S rRNA microbial diversity were conducted by Shanghai Majorbio Biomedical Technology Co., Ltd. Total bacterial DNA was extracted from fecal samples using a DNA extraction kit (Qiagen, Hilden, Germany), and DNA concentration and purity were assessed with a NanoDrop 2000. The hypervariable regions V3 and V4 of the bacterial 16S rRNA gene were amplified using primers 515F (5′‐GTGCCAGCMGCCGCGGTAA‐3′) and 806R (5′‐GGACTACHVGGGTWTCTAAT‐3′). The resulting sequencing library was sequenced on the Illumina NovaSeq platform (Novagene, CNH). Raw sequencing reads were quality controlled using fastp (https://github.com/OpenGene/fastp, version 0.20.0) and assembled with FLASH (http://www.cbcb.umd.edu/software/flash, version 1.2.7). The DADA2 plugin in the QIIME2 pipeline, with default parameters, was used to denoise the quality‐controlled and assembled sequences to generate Amplicon Sequence Variants (ASVs). A phylogenetic tree was constructed using the SEPP algorithm with default settings and the Silva 138 database as a reference. All data analyses were performed on the Majorbio I‐Sanger cloud platform (https://cloud.majorbio.com/).

### Viability Assay of *L.intestinalis*

*L. intestinalis* was cultured in MRS medium under microaerobic conditions at 37 °C and exposed to AS at concentrations of 50 mg L^−1^ (L‐AS), 100 mg L^−1^ (H‐AS), or to each of the ten main components of AS at 50 mg L^−1^. OD600 values were measured at various co‐cultivation time points using a microplate reader.

### Fecal Bile Acids Test

The BAs standard was accurately weighed and dissolved in methanol to prepare a stock solution with a final concentration of 1000 µg mL^−1^. This stock solution was diluted with 30% methanol to generate ten standard curve points. All stock and working standard solutions were stored at −20 °C. An appropriate amount of fecal sample was placed in a 2 mL centrifuge tube, and 400 µL of cold extraction solvent (methanol) was added. The mixture was vortexed for 60 s to ensure thorough grinding. Samples were then ultrasonicated at room temperature for 30 min and centrifuged at 12000 r min^−1^ at 4 °C for 10 min to remove proteins. Subsequently, 300 µL of the supernatant was transferred, mixed with 600 µL of water, and filtered through a 0.22 µm membrane. The filtrate was transferred to a sample vial for metabolomics analysis using the SCIEX QTRAP 5500 ultra‐performance liquid chromatography‐tandem mass spectrometry (UPLC‐MS/MS) system. Chromatographic and mass spectrometry conditions were as follows: an ACQUITY BEH C18 column (100 mm × 2.1 mm, 1.7 µm) was maintained at 40 °C. The mobile phase consisted of A (0.1% aqueous formic acid solution) and B (a mixture of acetonitrile and isopropanol containing 0.1% formic acid) at a flow rate of 0.4 mL min^−1^, with an injection volume of 2 µL. Mass spectrometry parameters included spray voltages of 3500 V (positive mode) and 2800 V (negative mode), sheath gas flow at 40 psi, auxiliary gas flow at 10 psi, a temperature of 400 °C, and an ion transfer tube temperature of 320 °C. The UHPLC‐Q Exactive Mass spectrometer (Thermo Fisher Scientific) was used with an electrospray ionization (ESI) source. The resolution for the first (MS1) full scan was set at 70000, and the second (MS2) fragmentation resolution was 17500, with normalized collision energies of 20, 40, and 60 eV.

### Cell Culture

The IEC‐6 (RRID:CVCL_0343, Catalog No.: CRL‐1592), HT‐29 (RRID:CVCL_0320, Catalog No.: HTB‐38), and Caco‐2 (RRID:CVCL_0025, Catalog No.: HTB‐37) cell lines were obtained from the American Type Culture Collection (ATCC) on July 4, 2023, and stored in liquid nitrogen. All cell lines were confirmed to be free of contamination. Cells were cultured following the supplier's protocols. Synchronization was performed in serum‐free medium for 12 h, followed by treatment with UDCA (20 µm, MCE #HY‐13771) with or without GW4064 (1 µm, MCE #HY‐50108) for 24 h. RNA was subsequently extracted using TRIzol.

### DNA Extraction and Bacteria Quantification

Bacterial DNA was extracted from mice small intestine contents, intestinal mucosa, and feces using a bacterial genomic extraction kit (Cat: D1600, Solarbio, China). DNA expression levels were measured with the SYBR Green Premix Pro Tag HS qPCR Kit (Code No. AG11701, Accurate Biotechnology Co., Ltd, China) on the Roche LightCycler 96 system (Basel, Switzerland). Relative abundance was determined using the ‐ΔCt method, employing universal bacterial 16S as the internal reference gene.

### Statistical Analysis

Statistical comparisons between two groups were performed using either the parametric Student's t‐test or the non‐parametric Mann‐Whitney U test, based on data distribution. For analyses involving more than two groups, either the Kruskal‐Wallis test or one‐way analysis of variance (ANOVA) was applied. When appropriate, P‐values were adjusted for multiple testing using the Benjamini‐Hochberg false discovery rate (FDR) method with a threshold of 0.1. All statistical analyses were conducted using GraphPad Prism 9 (GraphPad, San Diego, California, USA) or R software. Statistical significance was defined as a P‐value less than 0.05.

## Conflict of Interest

The authors declare no conflict of interest.

## Supporting information



Supporting Information

## Data Availability

The original sequencing data is available in the Sequence Read Archive at the National Center for Biotechnology Information (NCBI) under accession number PRJNA1297997. Cohort data for different age groups is obtained from the publicly available Metabolomics Workbench database, Study ID ST001851.
