# Supplementary material for: Medicago Sativa L. Saponin‐Driven Lactobacillus Intestinalis Restores Intestinal Stemness in Naturally Aged Mice via the Bile Acid‐FXR‐Wnt Signaling Axis
Source: Adv Sci (Weinh). 2025 Oct 27;13(4):e15370. doi: 10.1002/advs.202515370 (PMC12822443; doi:10.1002/advs.202515370)
Supplement: Supplementary file 1 — Supporting Information [file ADVS-13-e15370-s001.docx]

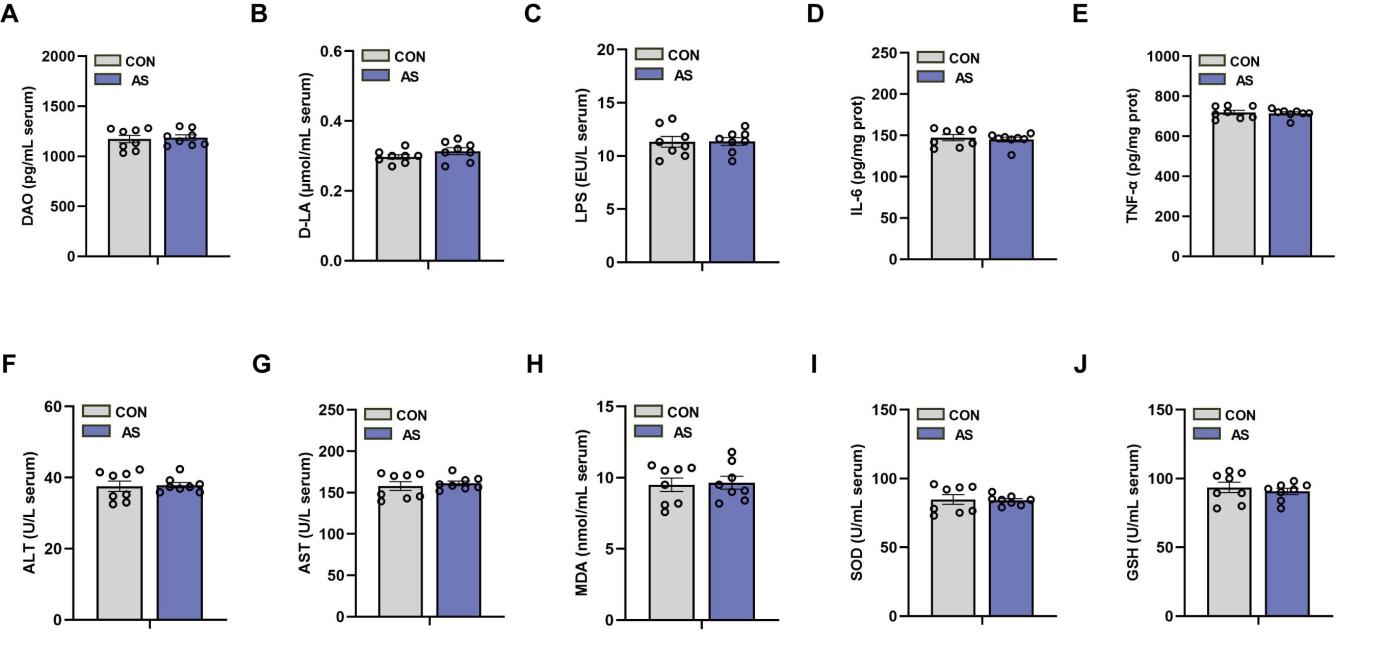


Figure.S1 The effects of AS on intestinal toxicity and hepatic toxicity in naturally aging mice. (A-E) Intestinal toxicity, including serum DAO, serum D-LA, serum LPS, levels of IL-6 in small intestine tissue, and levels of TNF-α in small intestine tissue. (F-J) Hepatic toxicity, including serum ALT, AST, MDA, SOD, and GSH. Data were statistically analyzed using a two-tailed Student's t-test and are presented as mean ± SEM. Statistical significance is indicated by *p < 0.05 and **p < 0.01.


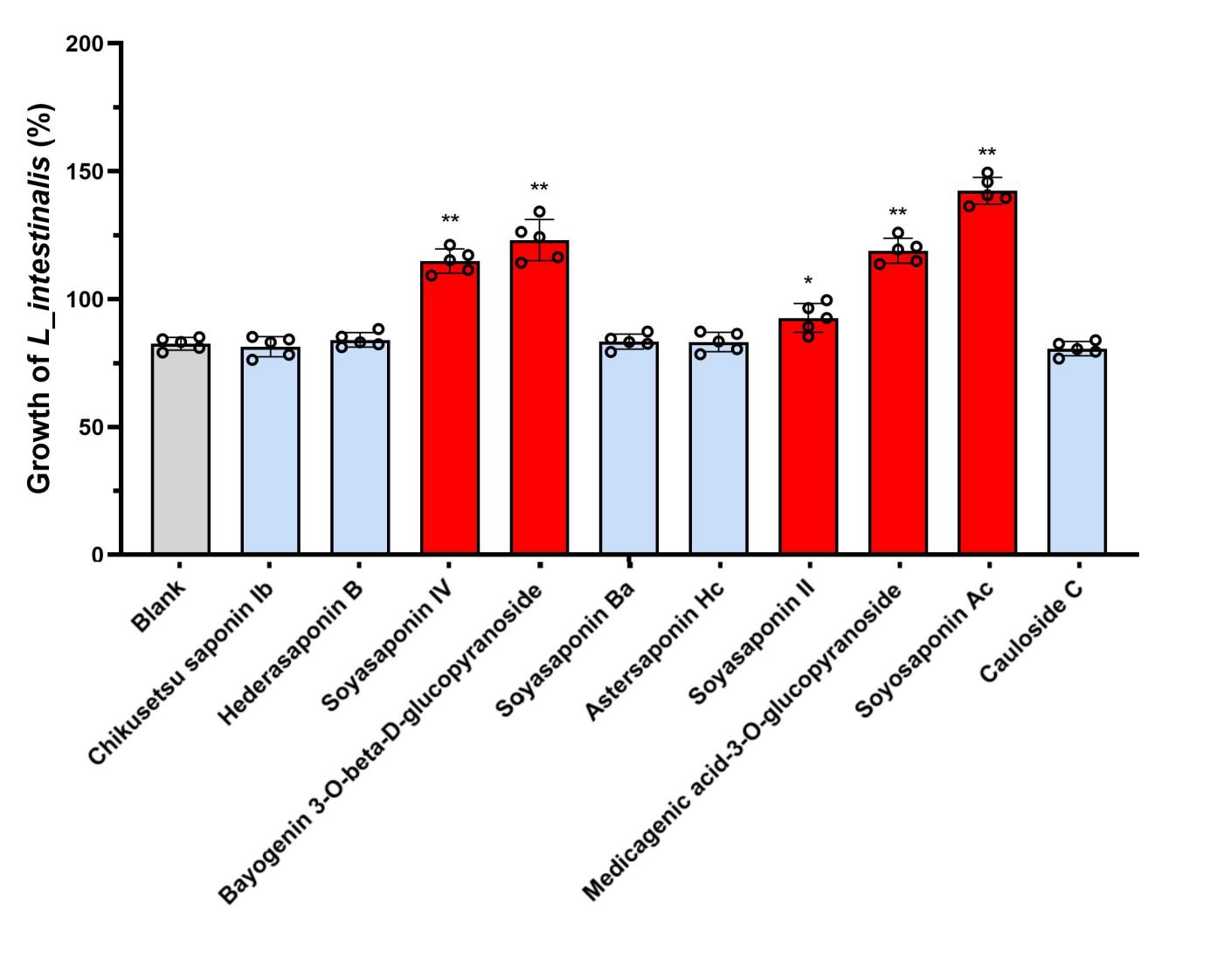


Figure.S2 Co-cultivation of the 10 main components of AS with *L. intestinalis*. Data were statistically analyzed using a two-tailed Student's t-test and are presented as mean ± SEM. Statistical significance is indicated by *p < 0.05 and **p < 0.01.


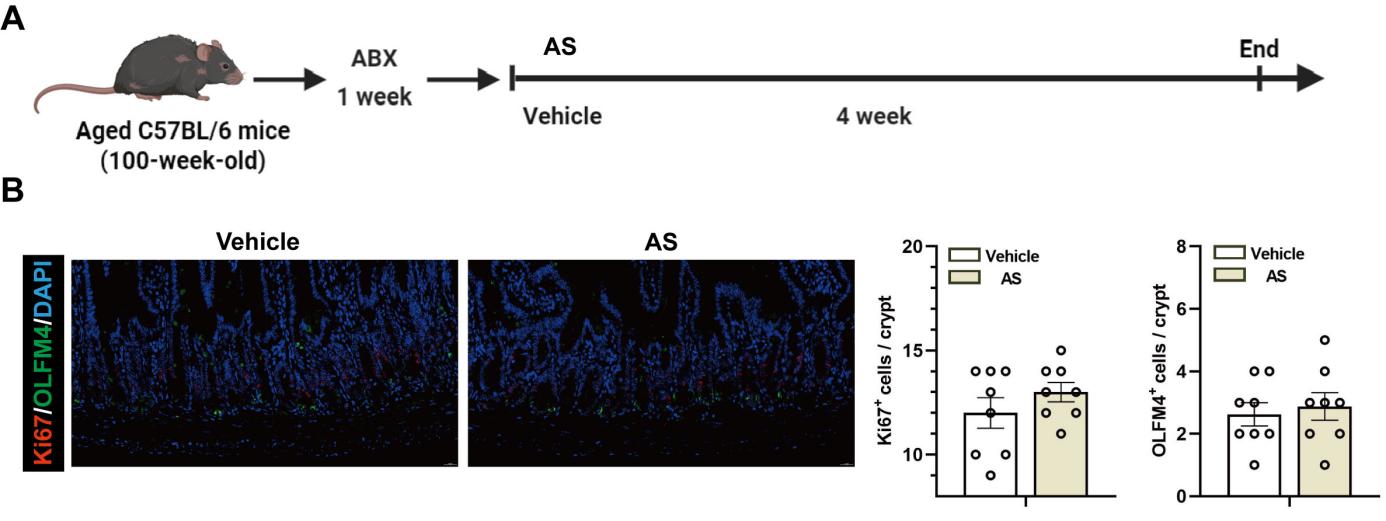


Figure S3 Effect of antibiotic pre-treatment on the expression of Ki67 and OLFM4 in the intestines of naturally aged mice. (A) Experimental design of the treatment protocol for naturally aged C57BL/6 mice (100 weeks old), which includes administering antibiotics (ABX) for one week followed by four weeks of solvent or AS treatment. (B) Representative images and quantitative values of immunofluorescence staining for the cell proliferation marker Ki67 (red) and the ISCs marker OLFM4 (green) in the small intestine. Scale bar 20 μm. Data were statistically analyzed using a two-tailed Student's t-test and are presented as mean ± SEM. Statistical significance is indicated by *p < 0.05 and **p < 0.01.


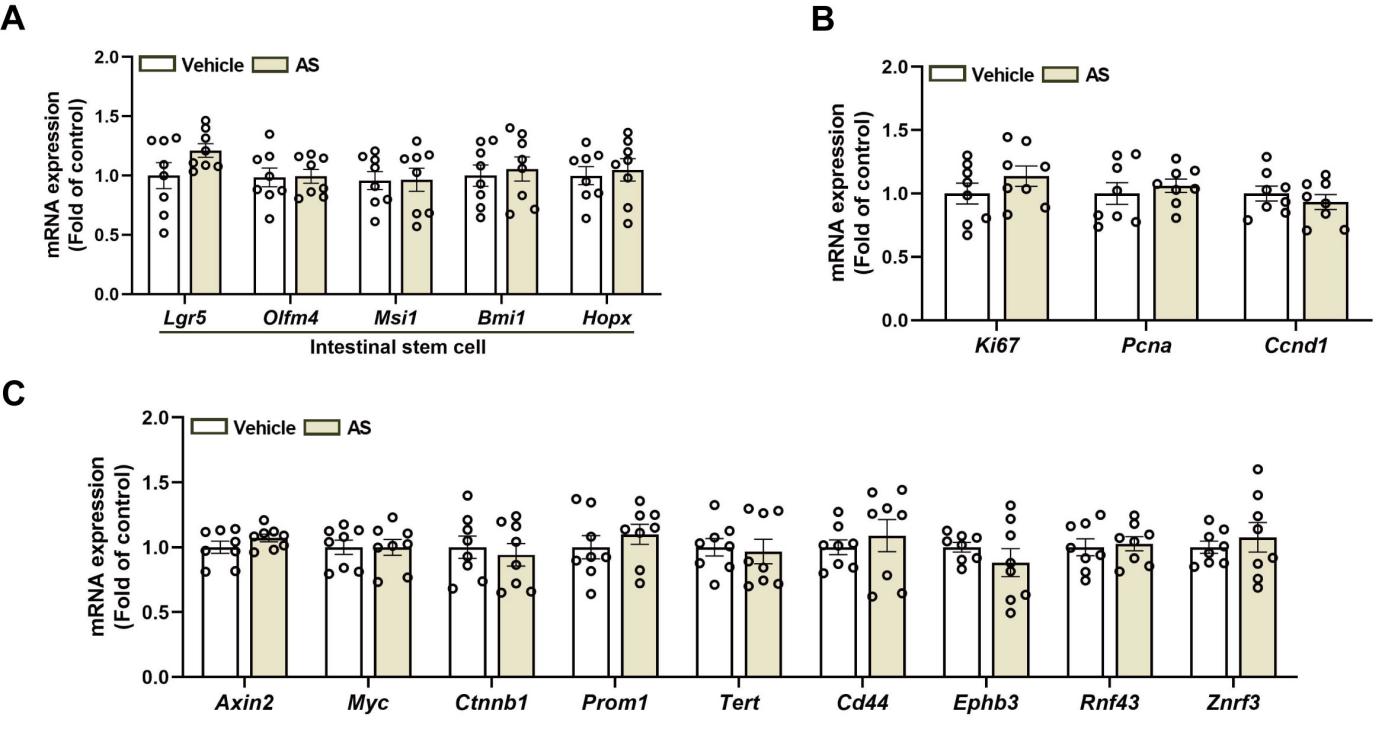


Figure S4 Effects of antibiotic pre-treatment on the expression of intestinal ISCs, cell proliferation markers, and Wnt signaling mRNA in naturally aged mice. (A) Relative expression levels of cell proliferation marker mRNA. (B) Relative expression levels of cell proliferation marker mRNA. (C) Relative mRNA expression levels of Wnt signaling pathway target genes in mouse jejunal tissue. Data were statistically analyzed using a two-tailed Student's t-test and are presented as mean ± SEM. Statistical significance is indicated by *p < 0.05 and **p < 0.01.


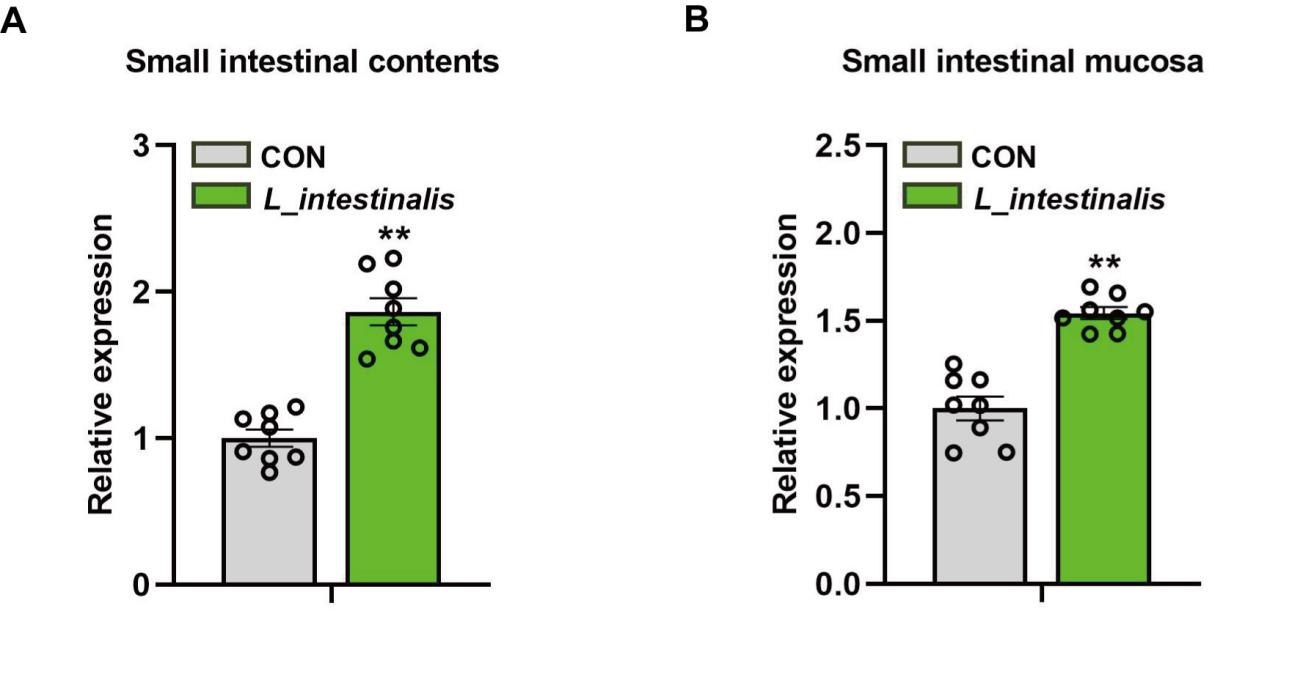


Figure.S5 Detecting *L. intestinalis* in small intestinal contents (A) and small intestinal mucosa (B) using RT-qPCR and specific bacterial primers. Data were statistically analyzed using a two-tailed Student's t-test and are presented as mean ± SEM. Statistical significance is indicated by *p < 0.05 and **p < 0.01.


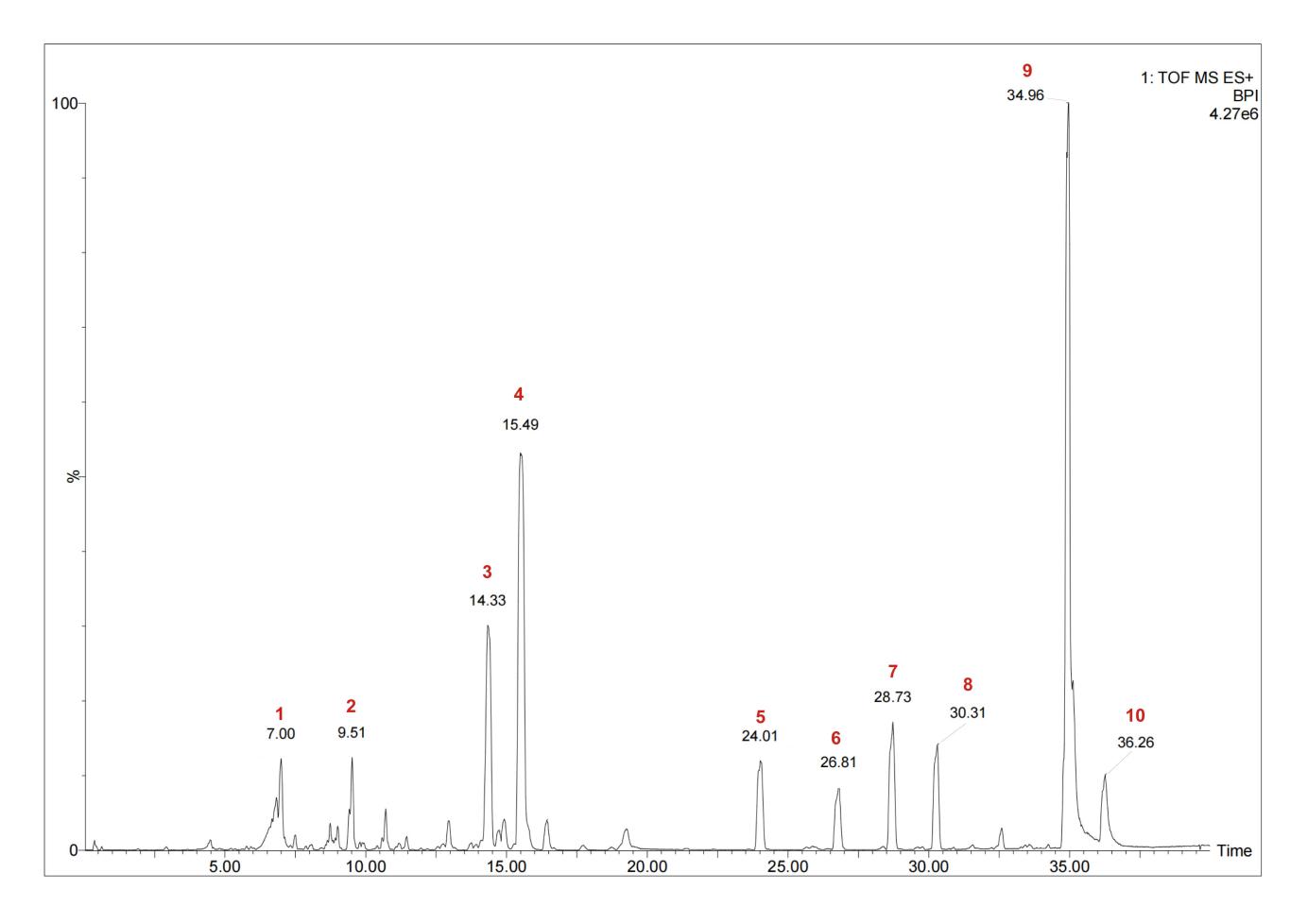


Figure.S6 Cation chromatogram of AS

**Table S1.** Top 10 peak areas of saponins compounds in AS

| NO. | t_R_ (min) | Molecular formula | Identification results | Molecular weights | Observed m/z | Adduct | PubChem CID |
| --- | --- | --- | --- | --- | --- | --- | --- |
| 1 | 7.00 | C_47_H_74_O_18_ | Chikusetsu saponin Ib | 927.08 | 966.16 | +K | 77916057 |
| 2 | 9.51 | C_59_H_96_O_25_ | Hederasaponin B | 1205.40 | 1206.37 | +H, +Na | 21626480 |
| 3 | 14.33 | C_41_H_66_O_13_ | Soyasaponin IV | 767.00 | 767.95 | +H | 24721354 |
| 4 | 15.49 | C_36_H_58_O_10_ | Bayogenin 3-O-beta-D-glucopyranoside | 650.80 | 673.81 | +H, +Na | 14031163 |
| 5 | 24.01 | C_48_H_78_O_19_ | Soyasaponin Ba | 959.10 | 977.14 | +Na, +H, +NH_4_ | 91973815 |
| 6 | 26.81 | C_57_H_90_O_26_ | Astersaponin Hc | 1191.30 | 1214.28 | +Na | 162890793 |
| 7 | 28.73 | C_47_H_76_O_17_ | Soyasaponin II | 913.10 | 952.18 | +K | 443614 |
| 8 | 30.31 | C_36_H_56_O_11_ | Medicagenic acid-3-O-glucopyranoside | 664.80 | 665.82 | +H, +Na | 162084 |
| 9 | 34.96 | C_67_H_104_O_32_ | Soyosaponin Ac | 1421.50 | 1440.55 | +NH_4_ | 137796351 |
| 10 | 36.26 | C_41_H_66_O_13_ | Cauloside C | 767.00 | 806.04 | +K | 13878151 |

**Table S2. Specific primers of related genes**

| Gene | Sequence (5’-3’) |
| --- | --- |
| Mist1-F | AGAGCAATGAGCGAGAGAGG |
| Mist1-R | GTGAGGGTCTCGATCTTGGA |
| Atoh1-F | GCCAGTTAGGAAGGCAACAG |
| Atoh1-R | ACAACGATCACCACAGACCA |
| Dll1-F | GTTGTCTCCATGGCACCTG |
| Dll1-R | TGCACGGCTTATGGTGAGTA |
| Tph1-F | GTCCTGTGGCTGGTTACCTC |
| Tph1-R | GCTCTGGAGTGTAGAGGGGA |
| Chga-F | CCCACTGCAGCATCCAGTT |
| Chga-R | CCGACTGACCATCATCTTTCTG |
| Gpr119-F | CCGTGGCTGATACCTTGATT |
| Gpr119-R | AGAGGCAGCTGCAGAAGAAG |
| Gpr120-F | AGAGGCTTACGCTGAGCTTG |
| Gpr120-R | GAAGGAAACCATGAGCAGGA |
| Prox1-F | TGAATCCCCAAGGTTCAGAG |
| Prox1-R | AAAGGCATCATGGCATCTTC |
| Lyz1-F | ATGGCTACCGTGGTGTCAAG |
| Lyz1-R | ATCCCATAGTCGGTGCTTCG |
| Defa24-F | TGTAGAGCAAGAGGCTGCAA |
| Defa24-R | CAGCATCAGTGGCCTCAGTA |
| Cd24a-F | CTTCTGGCACTGCTCCTACC |
| Cd24a-R | TACTTGGATTTGGGGAAGCA |
| Sox9-F | CGACTACGCTGACCATCAGA |
| Sox9-R | AGACTGGTTGTTCCCAGTGC |
| Spdef-F | TTGGATGAGCACTCGCTAGA |
| Spdef-R | AAAAGCCACTTCTGCACGTT |
| Tff3-F | GATTACGTTGGCCTGTGTCC |
| Tff3-R | CAGGGCACATTTGGGATACT |
| Agr2-F | CAAATCTGGAGCCAAAAAGG |
| Agr2-R | CCATCAAGGGTCTGTTGCTT |
| Manf-F | CCACCATATCCCTGTGGAAA |
| Manf-R | CGTCCAGGATCTTCTTCAGC |
| Muc2-F | CTGACCAAGAGCGAACACAA |
| Muc2-R | CATGACTGGAAGCAACTGGA |
| Muc3-F | GCTGGCTTTCATCCTCCACT |
| Muc3-R | GCTGTCGTCTTGGGTGCTAT |
| Muc4-F | GAGGGCTACTGTCACAATGGAGGC |
| Muc4-R | AGGGTTCCGAAGAGGATCCCGTAG |
| Muc13-F | CTGCAACCCTAACCCCTGTA |
| Muc13-R | CGTTCCTTTCACACATGACG |
| Fabp1-F | ATTCATGAAGGCAATAGGTCTG |
| Fabp1-R | TCATGCACGATTTCTGACAC |
| Fabp2-F | GTGGAAAGTAGACCGGAACGA |
| Fabp2-R | CCATCCTGTGTGATTGTCAGTT |
| Apoc3-F | CGTAGGTGCCATGCAGCCCC |
| Apoc3-R | CAGCTCGGGCAGATGCCAGG |
| Alpi-F | ATCATCTTCCTGGGAGACGG |
| Alpi-R | CGCCGATGGTCTTGTAGTTG |
| Ki67-F | ATCCAGATGATGGAGCCAAG |
| Ki67-R | ATTTCTGCAGCTGGTTTGCT |
| Ccnd1-F | CCAGCTCCTGTGCTGCGAAG |
| Ccnd1-R | CATGGATGGCACAATCTCCT |
| Lgr5-F | CAGGTCAATACCGGAGCGAG |
| Lgr5-R | GCGAGGCACCATTCAAAGTC |
| Olfm4-F | GCCAGATCTTGGCTCTGAAG |
| Olfm4-R | GCCAGTTGAGCTGAATCACA |
| Msi1-F | GAGGACTCAGTTGGCAGACC |
| Msi1-R | CGCCTGGTCCATGAAAGTGA |
| Bmi1-F | TGCTGGAGAGCTGGAAAGTG |
| Bmi1-R | GTGAGGGAACTGTGGGTGAG |
| Hopx-F | ACCAGGTGGAGATCCTGGAGTA |
| Hopx-R | CCAGGCGCTGCTTAAACCAT |
| Pcna-F | CAAAGACCTCATCAATGAGG |
| Pcna-R | GAGGTTCACGCCCATGGCTA |
| Axin2-F | CTCCCCACCTTGAATGAAGA |
| Axin2-R | ACT GGGTCGCTTCTCTTGAA |
| Myc-F | GCTGTTTGAAGGCTGGATTTC |
| Myc-R | GATGAAATAGGGCTGTACGGAG |
| Ctnnb1-F | CCCAGTCCTTCACGCAAGAG |
| Ctnnb1-R | CATCTAGCGTCTCAGGGAACA |
| Prom1-F | GAAAAGTTGCTCTGCGAACC |
| Prom1-R | TCTCAAGCTGAAAAGCAGCA |
| Tert-F | ACTCAGCAACCTCCAGCCTA |
| Tert-R | CATATTGGCACTCTGCATGG |
| Cd44-F | GTGGGCAGAAGAAAAAGCTG |
| Cd44-R | TGATGGTTCCTTGTTCACCA |
| Ephb3-F | CGTGAAAGTGGACACCATTG |
| Ephb3-R | CCAAGTAGAAGCCAGCCTTG |
| Rnf43-F | ATGTAACCTCGTGGGTCTGC |
| Rnf43-R | GCCAACTTTCTGCTCCACTC |
| Znrf3-F | AGAAGCCATCGACCAGCTAA |
| Znrf3-R | AAGTACTCGGTGGGTTGTCG |
| FXR-F | TGGGCTCCGAATCCTCTTAGA |
| FXR-R | TGGTCCTCAAATAAGATCCTTGG |
| SHP-F | GCACGATCCTCTTCAACCCA |
| SHP-R | CAGAAGGGTGCCTGGAATGT |
| FGF15-F | TGAGCCATCCAGTTGTGTCC |
| FGF15-R | CCACTGGAGAATTTGGGGCT |
| SHP-F (H) | CCCCAAGGAATATGCCTGCC |
| SHP-R (H) | TAGGGCGAAAGAAGAGGTCCC |
| FGF19-F (H) | CCAGAAGACAGGCAGTAGT |
| FGF19-R (H) | CTGGAGGGATTTGGGAAGG |
| GAPDH-F | GGAGAAACCTGCCAAGTATG |
| GAPDH-R | TGGGAGTTGCTGTTGAAGTC |
| *L. intestinalis*-F | ATCCGCTAGAAGCTGTGGAAA |
| *L. intestinalis* -R | AATCACCTGCATACACGGCT |
| *universal Eubacteria* 16s-F | CGGCAACGAGCGCAACCC |
| *universal Eubacteria* 16s-R | CCATTGTAGCACGTGTGTAGCC |

**Table S3. Antibodies used in this study for western blot**

| Antibody name | Company | RRID | product category | Dilution ratio |
| --- | --- | --- | --- | --- |
| MUC2 | Proteintech Group, Wuhan, China | AB_2880943 | Cat No. 27675-1-AP | 1:800 IF |
| ChgA | Proteintech Group, Wuhan, China | AB_2879259 | Cat No. 23342-1-AP | 1:500 IF |
| Ki67 | Proteintech Group, Wuhan, China | AB_2756525 | Cat No. 27309-1-AP | 1:500 IF |
| Lgr5 | Proteintech Group, Wuhan, China | AB_3086207 | Cat No. 30007-1-AP | 1:200 IF |
| β-catenin | Proteintech Group, Wuhan, China | AB_2086128 | Cat No. 51067-2-AP | 1:1000 WB |
| Active β-catenin | Cell Signaling | AB_2650576 | Cat No. 19807S | 1:1000 WB  1:200 IF |
| OLFM4 | Proteintech Group, Wuhan, China | AB_2918163 | Cat No. 28432-1-AP | 1:500 IF |
| FXR | Proteintech Group, Wuhan, China | AB_2879874 | Cat No. 25055-1-AP | 1:2000 WB  1:200 IF |
| β-Actin | Proteintech Group, Wuhan, China | AB_10700003 | Cat No. 20536-1-AP | 1:1000 WB |
| HRP Goat anti-Rabbit IgG | Active Motif, CA, USA | AB_3216397 | Cat No. 15015 | 1:1000 WB |
| HRP Goat anti-Mouse IgG | Active Motif, CA, USA | AB_3216396 | Cat No. 15014 | 1:1000 WB |
